# Expression of the MYB transcription factor gene *BplMYB46* affects abiotic stress tolerance and secondary cell wall deposition in *Betula platyphylla*

**DOI:** 10.1111/pbi.12595

**Published:** 2016-08-01

**Authors:** Huiyan Guo, Yucheng Wang, Liuqiang Wang, Ping Hu, Yanmin Wang, Yuanyuan Jia, Chunrui Zhang, Yu Zhang, Yiming Zhang, Chao Wang, Chuanping Yang

**Affiliations:** ^1^ State Key Laboratory of Tree Genetics and BreedingNortheast Forestry UniversityHarbinChina; ^2^ Department of Life Science and TechnologyMudanjiang Normal CollegeMudanjiangChina; ^3^ Key Laboratory of Fast‐Growing Tree Cultivating of Heilongjiang ProvinceForestry Science Research Institute of Heilongjiang ProvinceHarbinChina

**Keywords:** *Betula platyphylla*, *BplMYB46*, abiotic stress, secondary wall deposition

## Abstract

Plant MYB transcription factors control diverse biological processes, such as differentiation, development and abiotic stress responses. In this study, we characterized *BplMYB46*, an *MYB* gene from *Betula platyphylla* (birch) that is involved in both abiotic stress tolerance and secondary wall biosynthesis. BplMYB46 can act as a transcriptional activator in yeast and tobacco. We generated transgenic birch plants with overexpressing or silencing of *BplMYB46* and subjected them to gain‐ or loss‐of‐function analysis. The results suggest that BplMYB46 improves salt and osmotic tolerance by affecting the expression of genes including *SOD*,*POD* and *P5CS* to increase both reactive oxygen species scavenging and proline levels. In addition, BplMYB46 appears to be involved in controlling stomatal aperture to reduce water loss. Overexpression of BplMYB46 increases lignin deposition, secondary cell wall thickness and the expression of genes in secondary cell wall formation. Further analysis indicated that BplMYB46 binds to MYBCORE and AC‐box motifs and may directly activate the expression of genes involved in abiotic stress responses and secondary cell wall biosynthesis whose promoters contain these motifs. The transgenic *BplMYB46‐*overexpressing birch plants, which have improved salt and osmotic stress tolerance, higher lignin and cellulose content and lower hemicellulose content than the control, have potential applications in the forestry industry.

## Introduction

Plant growth and development are strongly influenced by various stresses, such as salinity, drought and extreme temperatures (Su *et al*., 2014). A large number of transcription factors (TFs) mediate stress responses in plants, including MYB (Oh *et al*., 2011), NAC (Mao *et al*., 2012), bZIP (Uno *et al*., 2000) and WRKY (Mare *et al*., 2004) family members. The MYB family is one of the largest families of TFs. *Arabidopsis* contains more than 198 *MYB* genes (Yanhui *et al*., 2006), cotton and *Populus* contain approximately 200 (Cedroni *et al*., 2003), maize contains 157 (Du *et al*., 2012a) and soybean contains 252 (Du *et al*., 2012b). The MYB family is divided into four classes, including 1R‐, R2R3‐, 3R‐ and 4R‐MYB proteins, according to the number of MYB domains (Dubos *et al*., 2010). R2R3‐MYB appears to be specific to plants and has been widely investigated (Kim *et al*., 2015; Li *et al*., 2006; Prouse and Campbell, 2012). MYBs bind to several *cis*‐acting motifs, including the following: MBSI (T/C)AAC(G/T)G(A/C/T)(A/C/T), which is involved in cell cycle control and resistance to low temperatures (Ma *et al*., 2009; Prouse and Campbell, 2012); MBSII (A/G)(G/T)T(A/T)GGT(A/G), which is involved in regulating secondary cell wall biosynthesis (Kim *et al*., 2012); MBSIIG, ACC(A/T)ACC(A/C/T), which is related to flavonoid biosynthesis (Grotewold *et al*., 1994); MYBCORE, CAGTTA and CTGTTG, which are associated with drought tolerance (Ithal and Reddy, 2004); and AC‐box, ACC(A/T)A(A/C)(T/C), which is related to secondary cell wall deposition (Zhong *et al*., 2013).

Plant MYBs regulate cell differentiation, organ formation, leaf morphogenesis, secondary metabolism and abiotic stress responses (Ambawat *et al*., 2013; Qi *et al*., 2015; Sun *et al*., 2015; Xu *et al*., 2015). For instance, *OsMYB4* overexpression enhances freezing tolerance in rice, and it also improves acclimation to cold and drought in transgenic apple (Pasquali *et al*., 2008; Vannini *et al*., 2004). TaMYB73 in wheat induces the expression of stress signalling genes and increases salinity tolerance in transgenic *Arabidopsis* (He *et al*., 2012). Overexpression of OsMYB48‐1 induces the expression of stress‐response genes and improves salinity and drought stress tolerance in rice (Xiong *et al*., 2014). The functions of MYBs in secondary wall biosynthesis have also been investigated. For example, *Arabidopsis* AtMYB46 and AtMYB83 function as master switches in a transcriptional network promoting secondary wall deposition that is regulated by the secondary wall‐associated NAC domain protein 1 (SND1) (McCarthy *et al*., 2009; Zhong *et al*., 2007). PtrMYB2, 3, 20 and 21 positively regulate secondary wall biosynthesis during wood formation in poplar trees (Zhong *et al*., 2013). Gain‐ and loss‐of‐function analysis showed that AtMYB61 regulates both stomatal aperture and the expression of genes involved in lignin deposition (Liang *et al*., 2005; Newman *et al*., 2004; Romano *et al*., 2012). *AtMYB52* is a target gene regulated by AtMYB46 during secondary wall biosynthesis. Overexpression of *AtMYB52* also improves drought tolerance by regulating the ABA signal transduction pathway, suggesting a possible connection between secondary wall deposition and ABA responses (Ko *et al*., 2009; Park *et al*., 2011). Although MYBs have been investigated in diverse biological systems, their functional roles have not been fully elucidated. In addition, many members of the large MYB transcription factor family have not yet been characterized.

In this study, we cloned and functionally characterized an MYB transcription factor, BplMYB46, from birch (*Betula platyphylla*), a pioneer tree species widely distributed from Europe to Asia that has important applications for the paper, building and furniture industries (Zhang *et al*., 2012). Our results show that overexpression of BplMYB46 improves salt and osmotic stress tolerance and mediates secondary cell wall deposition in transgenic birch. This study increases our knowledge of the crosstalk between the abiotic stress and secondary cell wall biosynthesis pathways and provides insights into the functions of MYB proteins.

## Results

### BplMYB46 is an R2R3‐type MYB protein

The MYB transcription factor gene *BplMYB46* (GenBank accession number: KP711284) was isolated from *B. platyphylla*. Multiple sequence alignments (Figure S1) and phylogenetic analysis (Figure S2) indicate that BplMYB46 belongs to the R2R3‐type MYB subfamily, with a highly conserved domain in the N‐terminus containing R2 and R3 functional regions. Members of this subfamily, including VvMYB46, PtMYB2, PtMYB21 and AtMYB46, function in secondary cell wall biosynthesis.

### Transactivating BplMYB46 localizes to the nucleus

**Figure 1:**
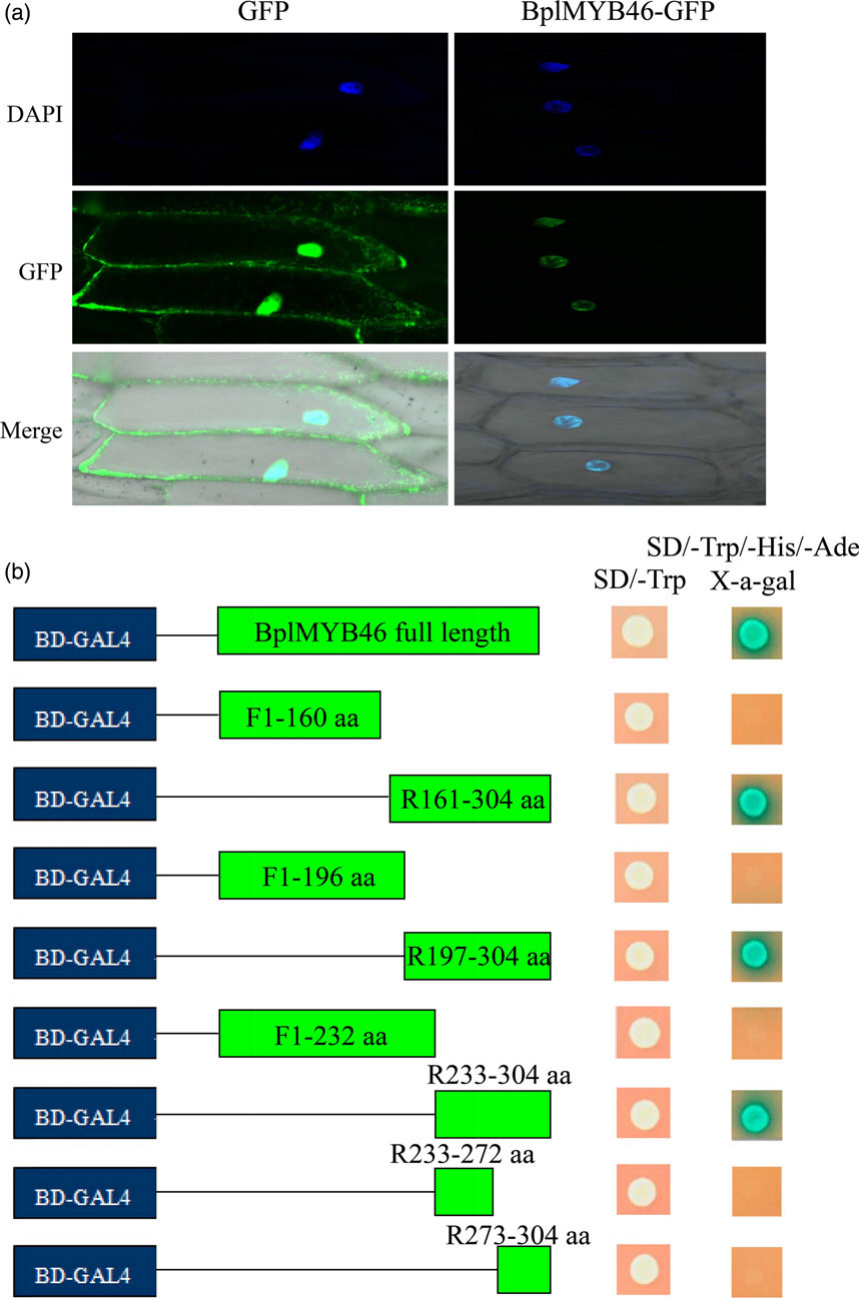
Subcellular localization and transactivation assay of BplMYB46. (a) The 35S : *BplMYB46*‐*GFP* fusion gene and 35S:*GFP* control plasmid were transformed into onion epidermal cells using particle bombardment. The transformed cells were imaged by confocal microscopy after transformation for 24 h. DAPI: DAPI staining of nucleus; GFP: GFP fluorescence detection; merge: the merged images of bright‐field, GFP and DAPI staining. (b) Various truncated sequences of the CDS of *BplMYB46* were fused in‐frame, respectively, to the GAL4 DNA‐binding domain in pGBKT7 and transformed into AH109 yeast cells. The transformed cells were plated onto SD/‐Trp (growth control) or SD/‐Trp/‐His/‐Ade/X‐a‐Gal medium. The pGBKT7 empty vector was used as a negative control.

We transformed 35S : BplMYB46‐GFP into onion epidermal cells by particle bombardment, using 35S : GFP as the control. Green fluorescent signals from the 35S: BplMYB46‐GFP transformed cells were detected in the nuclei, which were stained using DAPI. By contrast, 35S : GFP signals were uniformly distributed throughout the cell (Figure 1a), indicating that BplMYB46 is a nuclear protein.

To investigate whether BplMYB46 activated transcription and to identify the activation domain, a series of deletions of the BplMYB46 CDS were fused with the GAL4 DNA‐binding domain sequence in pGBKT7 (Clontech). The resulting constructs were, respectively, transformed into yeast cells for transcriptional activation analysis using the yeast two‐hybrid system (Y2H). Yeast cells harbouring the full CDS of BplMYB46 grew normally on SD/‐Trp/‐His/‐Ade/X‐α‐Gal medium, and x‐α‐gal was activated (Figure 1b), suggesting that BplMYB46 is a transcriptional activator. Furthermore, analysis of deletions with truncated CDS of BplMYB46 suggested that the transcriptional activation domain is located in a region from amino acid 233 to 304 in BplMYB46 (Figure 1b).

### Analysis of the expression of *BplMYB46*

**Figure 2:**
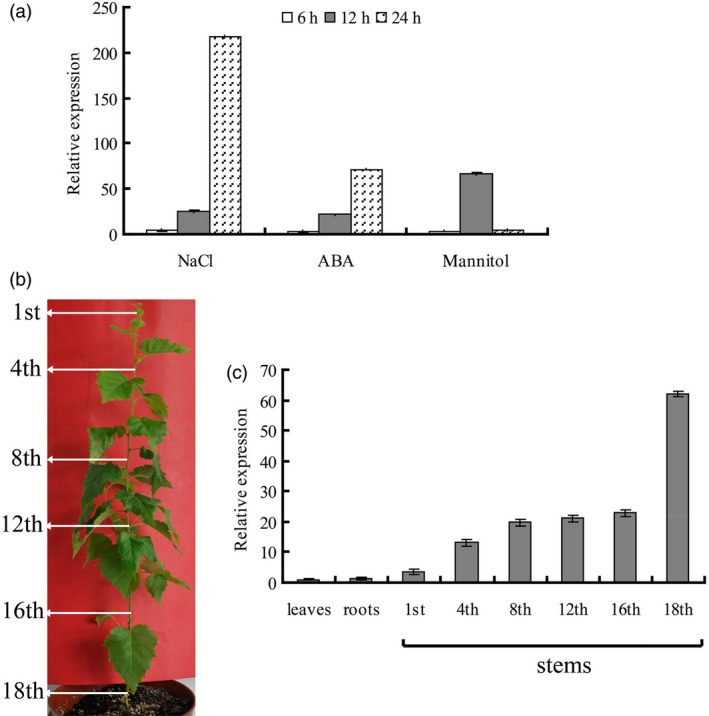
Expression patterns of *BplMYB46*. (a) Expression of *BplMYB46* in response to NaCl, ABA and mannitol treatment. Well‐watered plants were used as controls to normalize the expression level at each time point. (b) The positions of the birch stem internodes analysed for gene expression. Arrows indicate the different stem internodes used in the analysis. (c) *BplMYB46* mRNA levels in the roots, leaves and 1st, 4th, 8th, 12th, 16th and 18th stem internodes from 6‐month‐old birch plants. The *BplMYB46* mRNA level in birch leaves was set to 1 to normalize its transcription level to that of roots and stem internodes. The error bars indicate the standard deviation (SD) from three biological replicates.

To gain insight into the biological role of BplMYB46, we analysed the expression patterns of *BplMYB46* in response to NaCl, ABA and mannitol treatment using real‐time PCR. The results show that *BplMYB46* expression was induced by NaCl, ABA and mannitol after 6–24 h of treatment (Figure 2a). In addition, the expression patterns of *BplMYB46* in response to NaCl and ABA treatment were quite similar, that is induction after 6 to 24 h of treatment, reaching a peak at 24 h. Mannitol treatment increased *BplMYB46* expression at 6 to 24 h, with a peak at 12 h. We further examined *BplMYB46* expression in roots, leaves and various stem internodes of 6‐month‐old birch (Figure 2b). The expression of *BplMYB46* was higher in the stem internodes than in roots or leaves, with 60‐fold higher expression in the 18th internodes compared with leaves, whereas no significant difference in expression was observed between roots and leaves (Figure 2c). *BplMYB46* expression was lowest in the 1st internodes, with an increasing gradient from the stem tip to base. The highest expression level was observed in the 18th internodes of stems, with levels approximately 18‐fold those of the 1st internodes (Figure 2c). Therefore, *BplMYB46* is predominantly expressed in stems and is more highly expressed in mature tissue (base of stem) than in juvenile tissue (tip of stem).

### BpMYB46 binds to the MYBCORE and AC‐box motifs

**Figure 3:**
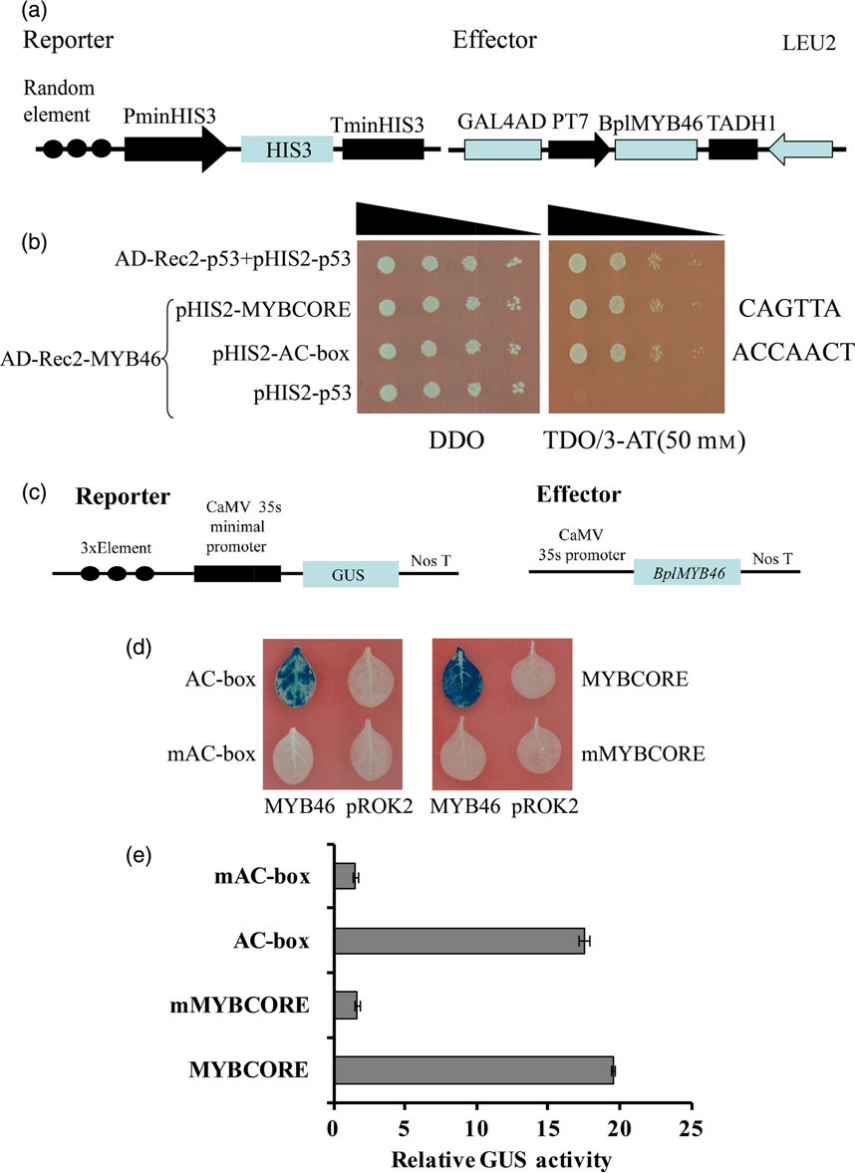
Analyses of BplMYB46 binding motifs. (a) Schematic diagram of the effector and reporter constructs used in Y1H analysis. (b) Analysis of binding of BplMYB46 to MYBCORE and AC‐box using Y1H. (c) Schematic diagram of the effector and reporter constructs used for coexpression in tobacco plants. (d, e) GUS staining (d) and GUS activity (e) Assays of the binding of BplMYB46 to the AC‐box and MYBCORE motifs in tobacco plants. The error bars indicate the standard deviation (SD) from three biological replicates.

Previous studies have shown that MYB proteins bind to the MYBCORE and AC‐box motifs (Ithal and Reddy, 2004; Zhong *et al*., 2013). To investigate the binding of BplMYB46 to these motifs, three tandem copies of these motif sequences (MYBCORE: CAGTTA; AC‐box: ACCACCT) were, respectively, cloned into pHIS2 and their interactions with BplMYB46 were determined using Y1H analysis (Figure 3a). The results indicate that yeast cells cotransformed with BplMYB46‐effector and different reporters grew on TDO/3‐AT medium, demonstrating that BplMYB46 also binds to the MYBCORE and AC‐box motifs (Figure 3b).

To further verify the above interactions identified by Y1H, the pROK2‐*BplMYB46* construct was used as an effector, and three tandem copies of MYBCORE and AC‐box, together with their mutants, were, respectively, fused with the minimal 35S promoter (−46 to +1) to drive the *GUS* reporter gene (Figure 3c). GUS activity was detected in tobacco leaves following cotransformation of lines harbouring pROK2‐*BplMYB46* with the MYBCORE and AC‐box motifs. However, very low GUS activity was detected in cotransformed lines harbouring pROK2‐*BplMYB46* and the respective mutant sequences (Figure 3d, e). MYBCORE is involved in drought stress responses (Ithal and Reddy, 2004), and the AC‐box plays a role in secondary wall biosynthesis (Zhong *et al*., 2013).

### Generation of transgenic birch plants with overexpression and knock‐down of *BplMYB46*

We generated 16 *BplMYB46*‐overexpressing (OE) and 15 RNAi‐silenced *BplMYB46* (SE) transgenic birch lines and examined the expression of *BplMYB46* using real**‐**time RT**‐**PCR. The transgenic and wild**‐**type (WT, nontransgenic) plants were generated from a single birch clone, which indicates that they have the same genetic background. In addition, the expression levels of endogenous *BplMYB46* in the transgenic and WT plants were similar. Therefore, the expression levels of the transgene *BplMYB46* and the functions of this gene could be investigated in the OE, SE and WT plants. Our results indicate that the expression of *BplMYB46* was significantly increased in the OE lines, with levels 3‐fold–38‐fold higher than those of WT, but were significantly reduced in the SE lines, with a 50%–91% decrease relative to WT (Figure S3). *BplMYB46*‐overexpressing transgenic lines 9 and 10 (termed OE9 and OE10, respectively), which showed moderate and high *BplMYB46* expression, respectively, were selected for further study. Two RNAi‐silenced *BplMYB46* lines, lines 3 and 15 (termed SE3 and SE15), which exhibited a high degree of silencing of *BplMYB46*, were also employed for further study.

### *BplMYB46* confers salt and osmotic stress tolerance

**Figure 4:**
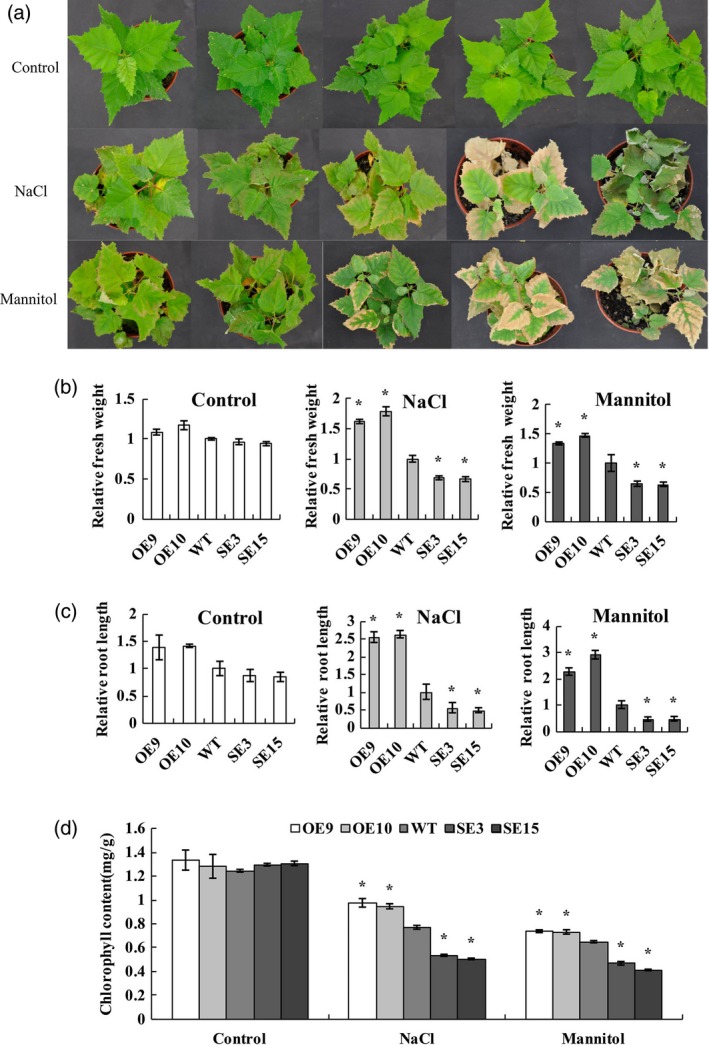
Salt and osmotic stress tolerance. (a) Comparison of growth phenotypes among OE, WT and SE birch plants under control, salt and osmotic stress conditions. (b–d) analysis of relative fresh weight (b), root length (c) and chlorophyll contents (d). Asterisk indicates *P* < 0.05. The error bars indicate the standard deviation (SD) from three biological replicates. ANOVA was used to determine statistically significant differences between results.

Soil‐grown transgenic birch plants, including OE, WT and SE lines, were exposed to salt or mannitol to evaluate their stress tolerance. There was no substantial difference in phenotype, growth rate, fresh weight or root length among OE, WT and SE lines under control conditions (Figure 4a), suggesting that *BplMYB46* does not affect the growth phenotype or growth rate of the plants. Under NaCl or mannitol treatment, compared with WT plants, both OE9 and OE10 displayed significantly higher growth rates, were greener and exhibited less wilting, in addition to having significantly higher fresh weights and root lengths. By contrast, lines SE3 and SE15 exhibited more severe leaf rolling and wilting, significantly reduced fresh weights and root lengths and the loss of green coloration compared with WT (Figure 4b, c). In addition, the chlorophyll contents were similar among OE, WT and SE lines under control conditions. However, under salt and osmotic stress conditions, compared with WT plants, both OE9 and OE10 had significantly higher chlorophyll levels, while SE3 and SE15 had significantly lower chlorophyll levels (Figure 4d). Overall, these results suggest that *BplMYB46* overexpression significantly improves abiotic stress tolerance in birch.

### *BplMYB46* affects reactive oxygen species (ROS) scavenging

**Figure 5:**
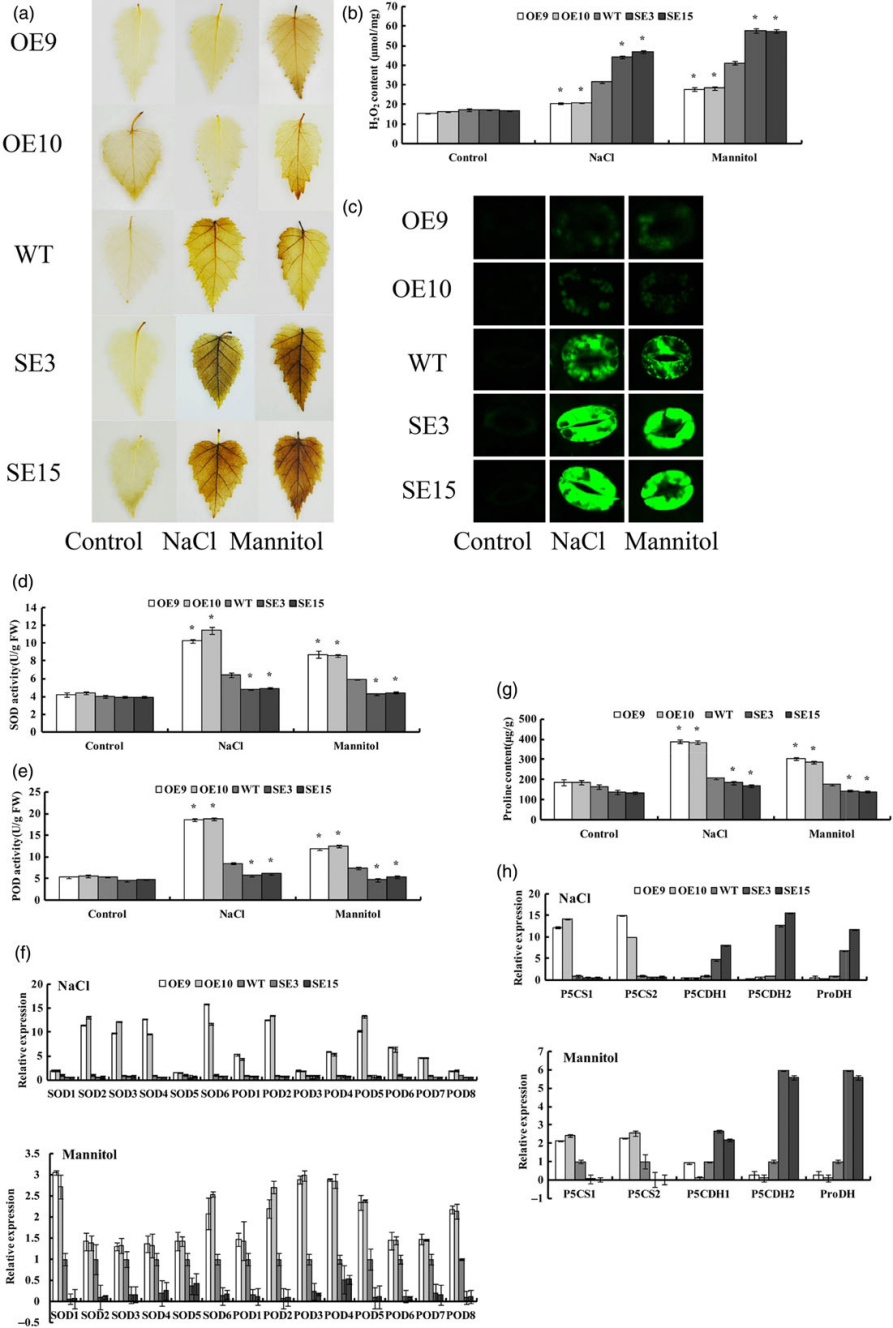
Detection of ROS scavenging and analysis of proline biosynthesis. (a) Detection of ROS using DAB *in situ* staining. (b) Assay of H_2_O_2_ levels. (c) Analyses of ROS production in intact guard cells by H2DCF‐DA staining. (d) SOD activity assay. (e) POD activity assay. (f) Expression analysis of *POD* and *SOD* genes under NaCl and mannitol treatment using qRT **‐** PCR. The expressions of the genes in WT plants were used as calculators to normalize their expressions in OE and SE lines. (g) Analysis of proline levels in OE, WT and SE lines. (h) Expression analysis of proline biosynthesis and degradation‐related genes following NaCl and mannitol treatment using qRT **‐** PCR. The expressions of the genes in WT plants were used to normalize their expressions in OE and SE lines. Asterisk indicates *P* < 0.05. The error bars indicate the standard deviation (SD) from three biological replicates. ANOVA was used to determine statistically significant differences between results.

In view of the key role of ROS in abiotic stress tolerance, we investigated whether *BpMYB46* affects ROS scavenging. We evaluated the levels of H_2_O_2_, a main ROS species, using DAB *in situ* staining. No obvious difference in DAB staining was observed among OE, WT and SE lines under control conditions. However, under NaCl or mannitol treatment conditions, compared with WT plants, OE9 and OE10 exhibited reduced DAB staining, whereas the two SE lines displayed strongly increased DAB staining, indicating that *BplMYB46* overexpression reduces H_2_O_2_ accumulation in plants (Figure 5a). We further measured H_2_O_2_ levels. Consistent with the DAB staining results, there was no difference among OE, WT and SE lines under control conditions. However, under salt and osmotic stress conditions, the H_2_O_2_ levels were highest in SE3 and SE15, followed by WT, whereas OE9 and OE10 had the lowest H_2_O_2_ levels (Figure 5b).

We also examined ROS levels in guard cells using DCFH‐DA fluorescence staining (Figure 5c). When exposed to NaCl or mannitol, ROS strongly accumulated in guard cells in the two SE lines, while both OE lines had substantially reduced ROS levels compared with WT plants. As ROS levels were significantly different in the OE, WT and SE lines when exposed to NaCl or mannitol, we also examined the activities of superoxide dismutase (SOD) and peroxidase (POD), the two major ROS scavenging enzymes in plants. The OE lines had significantly higher SOD and POD activity than the other lines, followed by WT, whereas the SE lines had significantly lower SOD and POD activity than the other lines (Figure 5d, E).

As the SOD and POD activities in these lines were altered in response to NaCl and mannitol treatment, we examined the expression of six *SOD* and eight *POD* genes in these lines. Under NaCl or mannitol treatment, the expression of all *SOD* and *POD* genes was highest in the OE lines, followed by WT and the SE lines (Figure 5f). These results indicate that BpMYB46 enhances ROS scavenging by affecting the expression of *SOD* and *POD*.

### *BplMYB46* affects proline biosynthesis

The proline levels were similar among OE, WT and SE lines under control conditions. However, under NaCl or mannitol treatment, the OE lines had significantly higher proline levels, followed by the WT and SE lines (Figure 5g). We further analysed the expression of genes related to proline biosynthesis (including *P5CS1* and *P5CS2)* and proline degradation (including *P5CDH* and *ProDH*). Compared with WT plants, the two *P5CS* genes were significantly more highly expressed in the OE lines but had reduced expression in the SE lines. Conversely, the proline degradation genes, including two *P5CDH* genes and one *ProDH* gene, were significantly down**‐**regulated in the OE lines but significantly up**‐**regulated in the SE lines compared with WT plants (Figure 5h).

### Overexpression of *BplMYB46* reduces cell death

**Figure 6:**
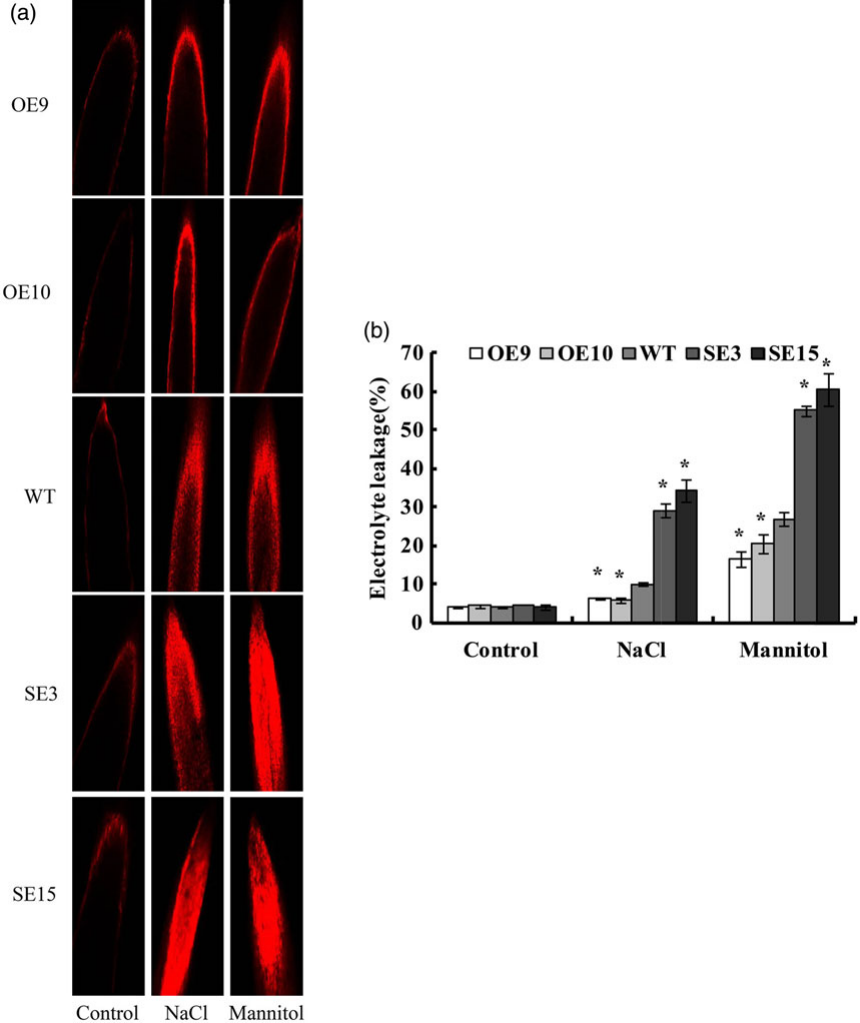
Analysis of cell death. (a) Assay of cell death among OE, WT and SE lines under NaCl and mannitol treatment using propidium iodide (PI) fluorescence staining. (b) Electrolyte leakage assay. Asterisk indicates P < 0.05. The error bars indicate the standard deviation (SD) from three biological replicates. ANOVA was used to determine statistically significant differences between results.

Propidium iodide (PI) staining reflects cell membrane damage based on fluorescence levels. We used PI staining to investigate cell death under NaCl or mannitol stress conditions. Compared with WT plants, the OE lines displayed relatively weak fluorescence, indicating reduced cell death, while the SE lines exhibited stronger fluorescence, suggesting severe cell death (Figure 6a). Consistently, the OE lines had the lowest electrolyte leakage rates, but the SE lines had the highest rates, when exposed to NaCl or mannitol (Figure 6b). Together, these results suggest that *BplMYB46* overexpression reduces cell death under salt and osmotic stress conditions.

### *BplMYB46* overexpression decreases water loss and reduces stomatal apertures

**Figure 7:**
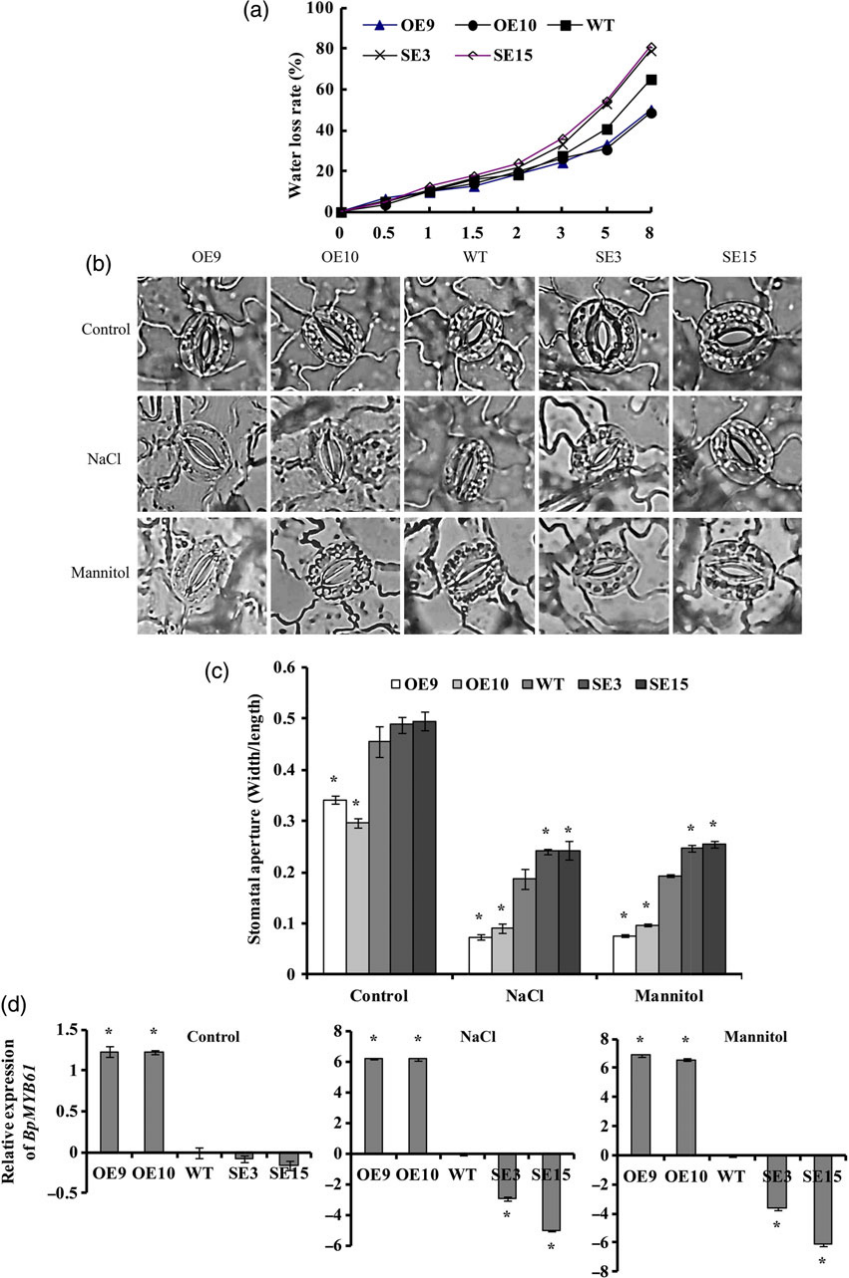
Analysis of water loss rate, stomatal aperture and *BpMYB61* expression in OE, WT and SE *BplMYB46* lines. (a) Water loss rates and (b, c) measurement of stomatal aperture under control conditions, 50 mm NaCl and 50 mm mannitol. (d) Expression of *BpMYB61* (GenBank accession number: KT344120) in OE, WT and SE *BplMYB46* lines under control conditions, 50 mm NaCl and 50 mm mannitol. Asterisk indicates *P* < 0.05. The error bars indicate the standard deviation (SD) from three biological replicates. ANOVA was used to determine statistically significant differences between results.

The OE lines showed significantly reduced water loss rates. The SE lines had elevated water loss rates compared with WT, with significantly higher water loss detected in detached leaves exposed to air (Figure 7a), indicating that *BplMYB46* overexpression reduces the transpiration rate in plants. Under control conditions, the stomatal apertures were smaller in the OE lines than in WT plants, whereas the stomatal apertures in the SE lines were slight larger than those of WT. Under salt and osmotic stress, the stomates in the OE lines were almost closed, in contrast to the stomates in the SE lines, which remained open (Figure 7b). In addition, the width/length ratios of stomatal apertures in the OE lines were significantly smaller than those of the WT and SE lines. However, under NaCl or mannitol stress, the width/length ratios of the stomatal apertures of the SE plants were significantly larger than those of the OE and WT plants (Figure 7c).

We also analysed the expression of *BpMYB61* (GenBank number: KT344120), a gene homologous to *Arabidopsis AtMYB61*, which encodes a protein that regulates stomatal aperture (Liang *et al*., 2005). Under control conditions, the expression of *BpMYB61* was significantly induced in the OE lines compared with WT plants, but no significant differences in *BpMYB61* expression were detected between the WT and SE lines. Under salt or osmotic stress conditions, the expression of *BpMYB61* was significantly induced in the OE lines. However, its expression was significantly reduced in the SE lines compared with WT plants (Figure 7d).

### *BplMYB46* affects vessel dimension and secondary wall thickening in fibres

**Figure 8:**
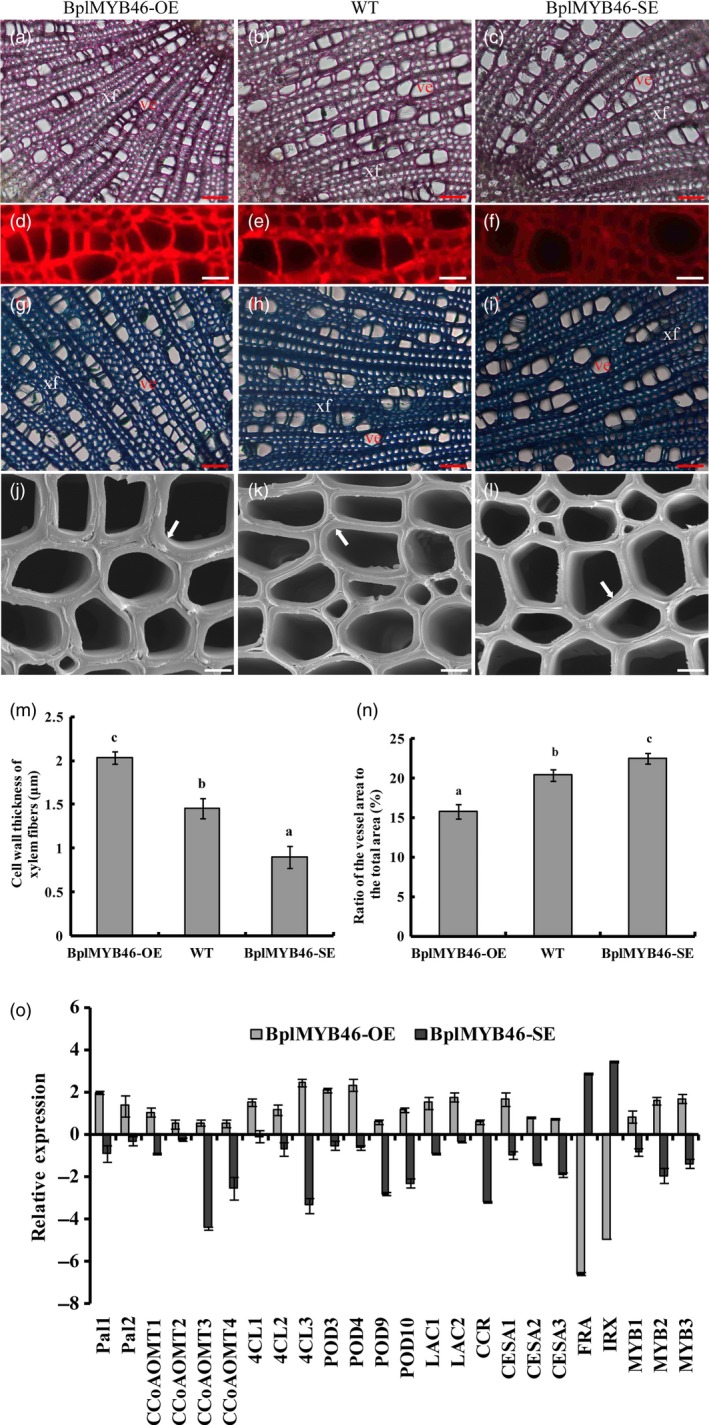
Microstructure of xylem vessels and fibres, and the expression of genes involved in secondary wall deposition. (a–c) Sections stained with phloroglucinol‐HCl (20×) in OE (a), WT (b) and SE plants (c); ve, vessel; xf, xylem fibre; bar = 50 μm. (d–f) Lignin autofluorescence in sections observed by microscopy (63×) in OE (d), WT (e) and SE (f), bar = 15 μm. (g–i) Sections stained with toluidine blue (20×) in OE (g), WT (h) and SE (i); ve, vessel; xf, xylem fibre, bar = 50 μm. (j–l) Sections analysed by scanning electron microscopy (3000×) in OE (j), WT (k) and SE (l); the arrow refers to the wall thickness of the xylem fibre, bar = 5 μm. (m) Secondary wall thickness of xylem fibres in OE, WT and SE. (n) Ratio of the vessel area to the total area (%) in OE, WT and SE. (o) Relative expression of genes related to secondary wall deposition in transgenic lines overexpressing *BplMYB46* (OE) and RNAi‐silenced *BplMYB46* (SE) lines. The sections were, respectively, analysed from the same stem positions of different plants of the same transgenic line. The error bars indicate the standard deviation (SD) from three biological replicates. ANOVA was used to determine statistically significant differences between results. a, b and c represent significant difference (*P* < 0.05) in OE, SE and WT birch plants. GenBank accession numbers: *Pal1,2*: KP711309 and KP711310; *CcoAOMT1‐4*: KP711311, KP711312, KT223488 and KT223489; *4CL1‐3*: KP711313, KP711314 and KP711315; *POD3,4,9,10*: KP711298, KP711299, KP711304 and KP711305; *LAC1,2*: KP711316 and KT223490; *CCR*: JQ783349; *CESA1‐3*: KP711317, KP711318 and KP711319; FRA: KU168419; *IRX*: KU168420; *MYB1‐3*: KP711285, KP711286 and KP711287.

We stained stem sections with phloroglucinol‐HCl and toluidine blue, finding that the number of vessels was higher in the OE lines than in WT plants, whereas the dimensions of the vessels in the OE lines were smaller than those of WT. Conversely, the number of vessels in the SE lines was reduced, and the vessel dimension was larger, compared to WT (Figure 8a–c, g–i). We measured the ratios of the vessel area to total area in anatomical sections of plants. Compared with WT plants, the ratio of vessel area to total area was lower in the OE lines but higher in the SE lines (Figure 8n).

Additionally, toluidine blue staining showed no substantial difference in secondary wall thickening in vessels among OE, WT and SE lines (Figure 8g–i). However, the secondary wall thickening of xylem fibres in WT plants was lower than that in OE lines but higher than that in SE lines (Figure 8j–l). Measurements using Image J software further indicated that the secondary wall thickness of xylem fibres in the OE lines was approximately 2 μm (±0.07), approximately 1.45 μm (±0.1) in WT and almost 0.9 μm (±0.1) in the SE lines (Figure 8m). These results suggest that BplMYB46 controls secondary cell wall thickness in fibres.

In the sections stained with phloroglucinol‐HCl, which stains lignin, the red staining was more intense in the OE lines but less intense in the SE lines compared with WT (Figure 8a–c). These results indicate that higher lignin deposition occurred in the OE lines than in WT, but less lignin was deposited in the SE lines than in WT. Detection of lignin autofluorescence (Figure 8d–f) indicated a stronger fluorescent signal in OE (Figure 8d) than in WT (Figure 8e) but a weaker signal in the SE lines (Figure 8f) than in WT, suggesting that BplMYB46 promotes lignin deposition. We also found significantly increased lignin content in the OE lines and significantly reduced content in the SE lines compared with WT, as determined by chemical analysis (Table 1), which is consistent with the results of lignin autofluorescence analysis (Figure 8d–f). The chemical analysis also suggested that the cellulose content in the OE lines was higher than that of WT. However, the hemicellulose content in the OE lines was dramatically reduced compared with WT (Table 1). These results indicate that BplMYB46 has a positive effect on lignin and cellulose content but a negative effect on hemicellulose content.

**Table 1:** Secondary cell wall composition of transgenic birch and WT plants

Line	Lignin (%)	Cellulose (%)	Hemicellulose (%)
BplMYB46‐OE	25.80 ± 0. 11	44.34 ± 0. 89	21.46 ± 1.3
Wild type	24.56 ± 0. 16	42.17 ± 0. 97	25.09 ± 1.0
BplMYB46‐SE	22.52 ± 0. 31	40.49 ± 0. 37	29.10 ± 2.8

Measurements were conducted on transgenic birch plants overexpressing *BplMYB46*, wild‐type (WT) plants and RNAi‐silenced *BplMYB46* plants. Values represent mean and standard deviation. All transgenic lines displayed significantly different values compared to WT (*P* < 0.05%). *n* = 11 (12 biological replicates).

### BplMYB46 affects the expression of secondary wall biosynthesis genes

We investigated the expression of the birch genes *phenylalanine ammonia lyase* (*PAL*), *caffeoyl‐CoA O‐methyltransferase* (*CCoAOMT*), *4‐coumarate‐coa ligase* (*4CL*), *POD*,* Laccase* (*LAC*), *cinnamoyl‐CoA reductase* (*CCR*), *cellulose synthase* (*CESA*), *fragile fibre* (*FRA*) and *irregular xylem* (*IRX*), which are homologous to lignin, cellulose and hemicellulose biosynthesis genes, via real‐time RT**‐**PCR. We also investigate the expression of the birch genes *BplMYB1, BplMYB2* and *BplMYB3*, which are homologous to *AtMYB42, AtMYB103* and *AtMYB52*, respectively; these genes are involved in secondary cell wall formation in *Arabidopsis*. The expression of genes related to lignin and cellulose biosynthesis was significantly induced in the OE lines but significantly down**‐**regulated in the SE lines. However, *FRA* and *IRX*, which are related to hemicellulose biosynthesis, were significantly down**‐**regulated in the OE lines but significantly up**‐**regulated in the SE lines (Figure 7o). These results suggest that BplMYB46 affects the expression of genes related to lignin, cellulose and hemicellulose biosynthesis in birch.

### ChIP and promoter–reporter analyses indicate BplMYB46 binding

**Figure 9:**
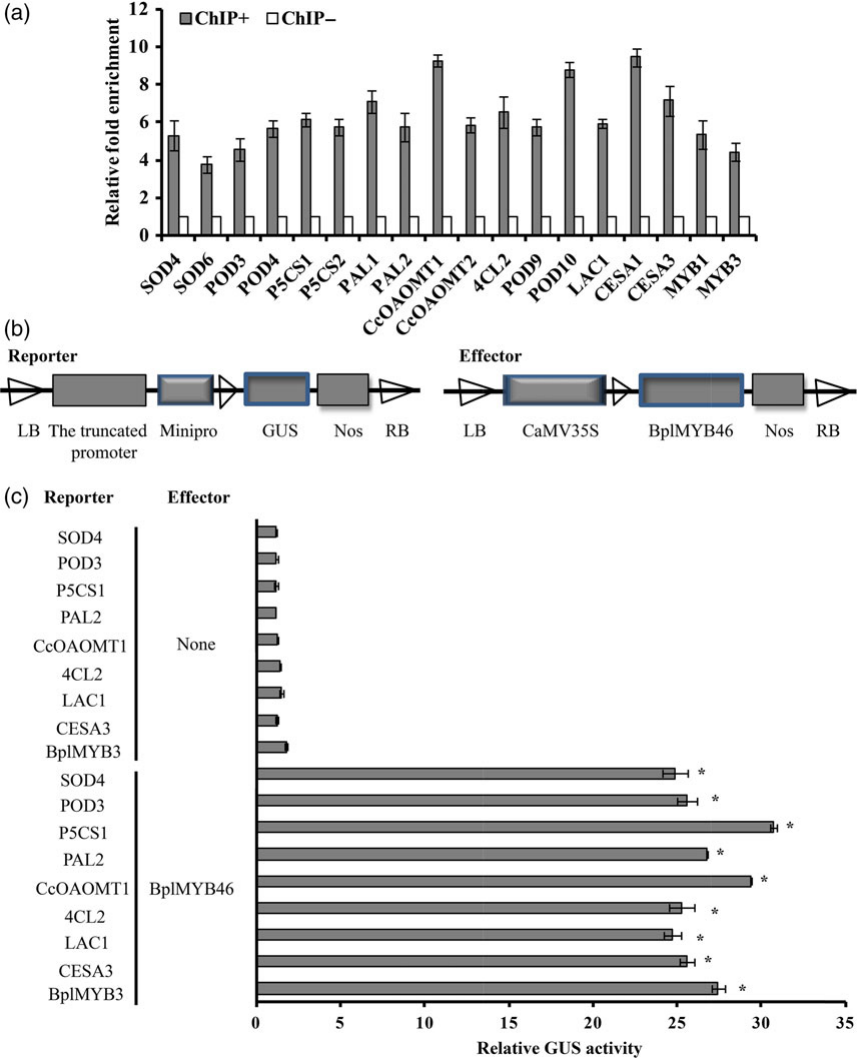
The regulation of target genes by BplMYB46, as determined by ChIP and GUS activity analyses. (a) Real‐time quantitative PCR analysis showing the enrichment of the promoter sequences of genes after chromatin immunoprecipitation. ChIP+: The sonicated chromatin was immunoprecipitated with GFP antibody; mock: the sonicated chromatin was immunoprecipitated with anti‐hemagglutinin (HA); input: the sonicated chromatin used as positive control. Three biological replicates were performed. Chromatin from whole seedlings was isolated from pROK2‐35S: BplMYB46‐GFP birch plants produced by *Agrobacterium tumefaciens*‐mediated transient transformation. The *tubulin* sequence was used as an internal control. After normalization against *tubulin*, the values of the enrichment of the promoter sequences of target genes in ChIP‐ were set to 1. The error bars indicate the standard deviation (SD) from three biological replicates. (b) Schematic diagram of the reporter and effector constructs used for coexpression in tobacco plants. (c) Relative GUS activity of the truncated promoters of genes. Asterisk indicates P < 0.05. The error bars indicate the standard deviation (SD) from three biological replicates. ANOVA was used to determine statistically significant differences between results. The GenBank accession numbers of promoters used in (a) and (c): KX373440–KX373458.

We performed ChIP analysis to determine whether BplMYB46 directly binds to the promoters of genes related to (1) abiotic stress (oxidative and osmotic stress), including *POD*,* SOD* and *P5CS* and (2) secondary wall deposition, including *PAL*,* CcoAOMT*,* 4CL*,* POD*,* LAC*,* CESA* and *MYB*. As BplMYB46 binds to MYBCORE and AC‐box motifs in genes related to abiotic stress and secondary wall deposition (Figure 9), and these two motifs are present in the promoters of target genes of BplMYB46 (Table S1), the primers for ChIP were designed to amplify the regions containing the MYBCORE and/or AC‐box motif. Quantitative ChIP‐PCR showed that the promoters of these putative target genes were significantly enriched (>threefold; Figure 9a) in the chromatin immunoprecipitated with GFP antibody (ChIP+) when compared with those in Mock sample (ChIP‐). These results indicate that BplMYB46 preferentially binds to the promoters of these genes, suggesting that BplMYB46 may directly regulate a set of genes that mediate abiotic stress responses and secondary wall biosynthesis.

To further verify that BplMYB46 binds to the promoters of this gene set as determined by ChIP, we cotransformed the pROK2‐*BplMYB46* and the truncated promoters containing the MYBCORE or AC‐box motif into tobacco leaves, and cotransformation with 35S : LUC was used to normalize the transformation efficiency values (Figure 9b). GUS activity measurement suggested that BplMYB46 can bind to the promoters of all genes investigated (Figure 9c).

## Discussion

Mette *et al*. (2000) showed that double‐stranded promoter RNA hairpins cause *trans*‐silencing of target genes triggered by methylation, which silences target genes more specifically than RNAi based on coding region. In the present study, as the genome of birch was not available early in our study, we employed RNAi‐based silencing of the promoter sequence to specifically knock down the expression of *BplMYB46*. The results show that the expression of *BplMYB46* was significantly reduced in the transgenic plants, with a minimum reduction of 90.77% (Figure S3), indicating that the RNAi‐based promoter used in our study was also functional in birch plants.

Although determining the copy number of a transgenic cassette is important for characterizing transgenic plants, the aim of the present study was to generate transgenic birch lines suitable for gain‐ and loss‐of‐function analysis of *BplMYB46*. Therefore, we directly determined the expression levels of *BplMYB46* in these plants, but we did not investigate the copy numbers of the cassette in OE and RNAi‐silenced lines. The results show that the expression of *BplMYB46* was significantly elevated in the OE lines and significantly reduced in the SE lines, indicating that the OE and SE lines were suitable for gain‐ and loss‐of‐function analysis, respectively.

In the present study, we used 200 mm NaCl or 300 mm mannitol solution for birch plant treatments. Sudden application of NaCl and mannitol in high concentration to plants would first cause osmotic shock rather than osmotic/salt stress, and followed by the imposition of an ionic shock (Shavrukov, 2013). Therefore, there is an overlap of changes in gene expression relating to ionic and osmotic responses, and these genes mainly involve in signal transduction, osmotic regulation, water loss and ionic component of salt stress response (Shavrukov, 2013).

In plants, Δ1‐pyrroline‐5‐carboxylate synthetase (P5CS) catalyses the rate‐limiting step in proline biosynthesis. *P5CS* overexpression greatly increases proline levels, while reducing *P5CS* expression abrogates the ability of the plant to accumulate proline (Deuschle *et al*., 2004). Two mitochondrial enzymes, pro‐dehydrogenase (ProDH) and P5C‐dehydrogenase (P5CDH), play sequential roles in catalysing proline degradation (Deuschle *et al*., 2004). In the current study, *BplMYB46* expression was positively correlated with the expression of *P5CS* but negatively correlated with the expression of *P5CDH* and *ProDH* (Figure 5h). Therefore, proline levels were positively correlated with *P5CS* expression but negatively correlated with *P5CDH* and *ProDH* expression. These results suggest that BplMYB46 induces proline biosynthetic genes and inhibits the expression of proline degradation genes, resulting in elevated proline levels and improved abiotic stress tolerance.

The transpiration rate in a plant is closely related to water stress tolerance. Our results indicate that *BplMYB46* expression reduced the transpiration rates of transgenic plants by controlling stomatal aperture to reduce water loss (Figure 7b, c). Indeed, overexpression of *AtMYB61* confers resistance to drought in transgenic *Arabidopsis* by reducing stomatal aperture (Liang *et al*., 2005). Therefore, to investigate whether BplMYB46 controls stomatal aperture by regulating the expression of genes homologous to *AtMYB61* in birch, we compared the sequences of BpMYB61 and AtMYB61, finding that they were highly homologous. We investigated the expression of *BpMYB61* in the OE, WT and SE lines. Our results suggest that BplMYB46 positively regulates the expression of *BpMYB61* (Figure 7d). These results suggest that BplMYB46 reduces water loss by positively regulating *BpMYB61*, which reduces the stomatal aperture to prevent water loss.

Overexpression of *PtrMYB3* and *PtrMYB20* in poplar increases the ectopic deposition of cellulose, xylan and lignin and regulates the expression of genes such as *CCoAOMT1*,* 4CL*,* FRA8*,* IRX8*,* IRX9*,* CesA4*,* CesA7* and *CesA8*, which are related to cellulose, xylan and lignin biosynthesis (McCarthy *et al*., 2010), suggesting that MYB transcription factors play an important role in secondary wall biosynthesis. In the current study, *BplMYB46* was more highly expressed in stems than in other tissues (Figure 2). Furthermore, overexpression of *BplMYB46* increased secondary wall thickening (Figure 8m). Knock**‐**down of *BplMYB46* reduced the secondary wall thickness in xylem fibres and decreased the lignin content (Figure 8m, Table 1). RT**‐**PCR analysis showed that overexpression and silencing BplMYB46 alter the expression of lignin, cellulose and hemicellulose biosynthesis‐related genes, including *PAL*,* CCoAOMT*,* 4CL*,* POD*,* CCR*,* LAC*,* CESA*,* FRA* and *IRX* (Figure 8o). In addition, ChIP‐qPCR shows BplMYB46 preferentially binds to these same promoters (Figure 9). These results strongly suggest that BplMYB46 may regulate a set of lignin, cellulose and hemicellulose biosynthesis‐related genes.

In the present study, we studied the bindings of BplMYB46 to promoters using ChIP method (Figure 9). However, the evidence of the bindings between the BplMYB46 and promoters was from plant lines with overexpression of the BplMYB46‐GFP fusion protein and therefore could be an artefact of ectopic expression. Additionally, a set of promoters were selected and used for ChIP‐PCR to investigate their bindings to BplMYB46, which does not provide a full picture of the impact of the native BplMYB46 on global steady state transcripts like RNA‐seq would. Therefore, to study the native BplMYB46 on global steady state transcripts in the future, ChIP‐Seq will be performed using BplMYB46 antibody to immunoprecipitate the chromatin bound by endogenous BplMYB46 in WT birch plants.

Birch plants are self‐sterile, meaning that it is impossible to generate homozygote plants by selfing. Additionally, birch trees do not reach the reproductive stage to produce seeds until they are more than 12 years old. Therefore, as it is impossible to produce homozygous transgenic plants by selfing, hemizygous transgenic plants were used in this study. In the future, when the transgenic plants reach the flowering stage, we plan to perform anther culture to generate haploid plants and to generate transgenic homozygous plants by performing chromosome doubling of the haploid plants.

## Experimental procedures

### Cloning and subcellular localization of BplMYB46

The cDNA sequence of *BplMYB46* was obtained from the birch transcriptome (Wang *et al*., 2014). The CDS of *BplMYB46* without the stop codon fused in‐frame to the N‐terminus of green fluorescent protein (GFP) was transformed into the pROK2 vector under the control of the CaMV 35S promoter (35S : MYB‐GFP; primer sequences are shown in Table S2). The GFP protein under the control of the 35S promoter was used as a control (35S : GFP). The 35S : MYB‐GFP and 35S : GFP constructs were introduced into onion epidermal cells by particle bombardment (Bio‐Rad laboratories, Inc. Hercules, California, USA). After incubation for 48 h, the transformed onion epidermal cells were stained with DAPI (100 ng/mL) and visualized under an LSM700 confocal laser microscope (Zeiss, Jena, Germany).

### Transactivation assay

The complete and various truncated versions of the CDS of *BplMYB46* were PCR amplified (using the primers listed in Table S3) and fused in‐frame to the GAL4 DNA‐binding domain in the pGBKT7 vector to generate the pGBKT7‐*BplMYB46* construct (Clontech laboratories, Inc. Mountain View, California, USA). The pGBKT7‐*BplMYB46* construct was transformed into AH109 yeast cells, which were incubated on SD/‐Trp or SD/‐Trp/‐His/‐Ade/X‐α‐Gal medium at 30 °C for 3–5 days.

### Plant materials and stress treatments

Birch seeds were planted in pots with a 12‐cm diameter containing a mixture of perlite/vermiculite/soil (1 : 1 : 4) in a greenhouse under controlled conditions (16/8 h light/dark, 25 °C and 70%–75% relative humidity). Each pot, which contained four seedlings, was thoroughly watered with deionized water every day. After 2 months, the plants were watered with 200 mm NaCl, 100 μm ABA or 300 mm mannitol solution, which was applied to the top of the soil. All seedlings were collected at the same time after treatment for 6, 12 or 24 h, and seedlings watered with deionized water were used as a control. Three independent biological replicates were performed, and each replicate included four seedlings. The roots, leaves and internodes of stems from 6‐month‐old birch plants were harvested, including the 1st, 4th, 8th, 12th, 16th and 18th stem internodes.

### Real‐time PCR

Total RNA was isolated from birch using the CTAB method (Chang *et al*., 1993) and treated with DNase I to remove DNA contamination. Total RNA was reverse transcribed into cDNA using a PrimeScript^™^ RT reagent Kit (Takara Bio Inc. Kusatsu, Shiga, Japan) for RT**‐**PCR analysis. Real**‐t**ime RT**‐**PCR was performed with a TransStart Top Green qPCR SuperMix kit (TransGen Biotech, Beijing, China) using the primer sequences listed in Table S4. The amplification procedure was conducted using the following parameters: 94 °C for 30 s; 45 cycles at 94 °C for 12 s, 58 °C for 30 s and 72 °C for 45 s; and 79 °C for 1 s for plate reading. Three independent experiments were performed in triplicate. The tubulin (GenBank accession number: FG067376) and ubiquitin (GenBank accession number: FG065618) genes were used as the internal controls. The relative expression level of each gene was calculated using the delta–delta CT method (Livak and Schmittgen, 2001).

### Examining the binding of BplMYB46 to the MYBCORE and AC‐box motifs using Y1H

Three tandem copies of MYBCORE (CAGTTA) and AC‐box (ACCAACT) were inserted into pHIS2 (Clontech) upstream of the reporter gene *HIS3,* respectively. The CDS of *BplMYB46* was cloned into pGADT7‐Rec2 (Clontech) as the effector (pGADT7‐*BplMYB46*). The constructs were cotransformed into Y187 cells, which were plated onto SD/‐Trp/‐His/ (DDO) and SD/‐Trp/‐His/‐Leu/ (TDO) medium supplemented with 50 mm 3‐AT (3‐amino‐1, 2, 4‐triazole) and incubated at 30 °C for 3–5 days. All primers used are listed in Table S5.

### Binding of BplMYB46 to motifs and promoters

Three tandem copies of MYBCORE, AC‐box, their mutated sequences and the truncated promoter were, respectively, fused with the 35S CaMV minimal promoter (−46 bp to +1) to drive the *GUS* gene in a modified pCAMBIA1301 vector (in which the 35S: hygromycin region was deleted) using the primers listed in Tables S6 and S7. The full CDS of *BplMYB46* was cloned into pROK2 under the control of the 35S promoter (35S : *BplMYB46*) as the effector. The effector was co‐transformed with each reporter into tobacco leaves by *Agrobacterium tumefaciens*‐mediated transient transformation (Ji *et al*., 2014). The firefly luciferase (*LUC*) gene driven by the 35S promoter was co‐transformed into tobacco leaves as a control for normalization of transformation efficiency. GUS and luciferase activities were determined as previously described (Gampala *et al*., 2001).

### Generation of plants with overexpression and silencing of *BplMYB46*

To silence *BplMYB46*, a truncated promoter of *BplMYB46* (223 bp in length) with sense and antisense sequences was inserted into pFGC5941 in forward and reverse direction to form an inverted repeat truncated promoter (pFGC5941*‐BplMYB46*;the primers were listed in Table S8). Transgenic birch lines were constructed using *Agrobacterium tumefaciens*‐mediated transformation to generate birch plants with overexpression (OE) or silencing (SE) of *BplMYB46*. Briefly, birch leaves were cut into small pieces, soaked in *Agrobacterium* suspension culture (OD_600_ = 0.5) for 5 min, washed three times with sterile water and incubated on woody plant medium (WPM + 2% [w/v] sucrose, pH 5.8) for 48 h. The plants were grown in selection medium containing WPM + 1.0 mg/L 6‐BA + 2% (w/v) sucrose + 50 mg/L kanamycin (for 35S : *BplMYB46* transformation) or 2 mg/L glufosinate (for pFGC5941*‐BplMYB46* transformation) + 600 mg/L carbenicillin, pH 5.8 to induce resistant callus. After the antibiotic‐resistant calli were regenerated, they were transferred to growth medium (WPM + 1.0 mg/L 6‐BA + 50 mg/L kanamycin or 2 mg/L glufosinate) for bud differentiation. Adventitious buds with 3–5 cm shoots were cut and transferred to root generation medium (WPM + 0.2 mg/L NAA + 50 mg/L kanamycin or 2 mg/L glufosinate).

### Analysis of stress tolerance phenotype

The transgenic OE, SE and WT birch plants were grown in pots containing a mixture of perlite/vermiculite/soil (1 : 1 : 4) under a 16‐/8‐h light/dark cycle at 25 °C and relative humidity of 75%. Each pot contained four plants. The seedlings were thoroughly watered with deionized water. After 2 months, the soil was directly watered with 200 mm NaCl or 300 mm mannitol every day for 10 d, and well‐watered plants served as controls. There were three replicates and four seedlings per replicates. The relative fresh weight, root length and chlorophyll content of each line were measured as described previously (Lichtenthaler, 1987).

### Histochemical analysis of stress response

Leaves detached at the third stem nodes of birch plants cultured for 2 months were incubated in a solution of 150 mm NaCl or 200 mm mannitol (or water for the control) for 6 h, stained with 3,3′‐diaminobenzidine (DAB, 1.0 mg/mL) (Zhang *et al*., 2011) and photographed. To observe ROS accumulation in intact guard cells, detached epidermal peels of leaves were incubated in deionized water (control) or a solution of 30 mm KCl and 10 mm MES‐KOH (pH 6.15) supplemented with 150 mm NaCl or 200 mm mannitol for 2 h. ROS were detected using 2,7‐dichlorofluorescin diacetate (H2DCF‐DA) as described previously (Zhang *et al*., 2011). For PI staining, the birch plants were incubated in a solution of deionized water (as a control), 150 mm NaCl or 200 mm mannitol for 24 h and 50 μg/mL of PI for 30 min, followed by three rinses with sterile water. The root tips were visualized under an LSM700 confocal laser microscope.

### Measurement of physiological parameters related to stress tolerance

OE, WT and SE *BplMYB46* plants grown in soil for 2 months were watered with a solution of 200 mm NaCl or 300 mm mannitol for 24 h. Well‐watered plants were used as controls. Superoxide dismutase (SOD) activity, peroxidase (POD) activity and electrolyte leakage were measured (Wang *et al*., 2012). Proline content was determined as previously described (Bates *et al*., 1973). H_2_O_2_ levels were measured as described previously (Sergiev *et al*., 1997). Each sample included at least three plants, and all experiments were conducted in triplicate.

### Water loss, stomatal aperture measurements and *BpMYB61* expression

The fresh weights (FW) of detached leaves were determined, and the leaves were then desiccated under normal atmospheric conditions. The leaves were weighed (desiccated weight) after exposure to air for 0.5, 1, 1.5, 2, 3, 5 and 8 h, dried overnight at 80 °C and their dry weights (DW) determined. The water loss rates (WLR) were calculated using the formula: WLR (%) = [(FW − desiccated weight)/(FW − DW)] × 100.

Epidermal peels were stripped from the leaves of 2‐month soil‐grown OE, SE and WT plants and floated in a solution of 30 mm KCl and 10 mm MES‐KOH (pH 6.15), followed by incubation for 2 h in the light at 22 °C to induce stomatal opening (Cheng *et al*., 2013). Then, 50 mm NaCl or 50 mm mannitol was added to the buffer solution. The samples were incubated for an additional 2 h. Stomatal apertures were photographed using light microscopy (Olympus BX43, Olympus Corporation, Shinjuku‐ku, Tokyo, Japan). The ratios of the widths and lengths of stomatal apertures under different treatments were calculated.

The expression of *BpMYB61*, a gene homologous to *AtMYB61*, encoding a protein that regulates stomatal aperture in *Arabidopsis*, was investigated in the OE, WT and SE *BplMYB46* lines under control conditions, 50 mm NaCl and 50 mm mannitol stress using the primers listed in Table S9.

### Histological analysis

Stems of 6‐month soil‐grown OE, WT and SE *BplMYB46* birch plants were fixed in FAA solution (70% ethanol: glacial acetic acid: formaldehyde; 90: 5: 5, v/v) and embedded in frozen sectioning medium (OCT; Thermo Scientific, Waltham, MA) to obtain 25‐μm‐thick stem base sections using a Microtome Cryostat (Thermo Scientific HM560). The stem sections were stained with phloroglucinol‐HCl and toluidine blue and examined by light microscopy (Zhong *et al*., 2006). Lignin autofluorescence was observed under a confocal laser microscope (Zeiss, Jena, Germany). For scanning electron microscopy (SEM), 0.2‐cm‐thick sections of 12‐month soil‐grown OE, WT and SE *BplMYB46* birch plants were obtained manually and observed under a scanning electron microscope (S‐4800, HITACHI, Tokyo, Japan). The ratio of vessel area to total area was measured from 12 anatomical sections representing each genotype after staining with phloroglucinol‐HCl. The secondary wall thickness of xylem fibres in the SEM micrographs was quantified in 45 cells using Image J software (http://rsbweb.nih.gov/ij/).

### Determination of secondary wall composition

The lignin, cellulose and hemicellulose levels of stems from the OE, WT and SE lines grown in soil for 12 months were determined according to the Klason procedure (Whiting *et al*., 1981) using an automatic fibre analyser (Ankom 2000i; Ankom, Macedon, NY). Twelve biological replicates were performed in this experiment.

### Expression analysis of *BplMYB46* target genes

For stress tolerance‐related gene expression analysis, birch plants grown for 2 months in soil were treated with 200 mm NaCl or 300 mm mannitol for 24 h; plants watered with deionized water only were harvested at the same time and used as controls. For wood formation‐related gene analysis, stems of the OE, WT and SE *BplMYB46* birch plants grown for 6 months in soil were harvested. All primers used are listed in Table S10. Three independent experiments were performed, with three biological replicates.

### ChIP assay

The 35S : BplMYB46‐GFP construct was transformed into birch plants for the ChIP assay. The ChIP assay was performed according to the published method (Haring *et al*., 2007). Briefly, after protein and chromatin were cross‐linked, the chromatin was sheared into 0.2–0.8 kb fragments by sonication, and 1/10 (volume) of each sample was used for the input control. Sonicated chromatin was immunoprecipitated with GFP antibody (Abmart) (ChIP+), and chromatin immunoprecipitated with antihemagglutinin (HA) antibody was used as a negative control (Mock). The antibody‐bound complex was precipitated with protein A + G agarose beads. The immunoprecipitated DNA was purified by chloroform extraction. The enrichment of the truncated promoters in immunoprecipitated samples were determined by real‐time PCR, and the *tubulin* sequence was used as an internal control. Three biological replications were performed. The primer sequences used for ChIP amplification are listed in Table S11.

### Statistical analysis

Histological indices involving stress responses and measurement of wood characters related to secondary wall deposition were analysed using ANOVA. All statistical analyses were performed using SPSS software (IBM. Chicago, IL, USA), version 18.0. Differential analysis of wood characters was performed using the DUNCAN method.

## Conflict of interest

The authors have no conflict of interest to declare.

## Supporting information


**Figure S1** Sequence alignments of BplMYB46 with other plant MYBs.
**Figure S2** Phylogenetic comparison of BplMYB46 with other plant MYBs.
**Figure S3** Relative expression of *BplMYB46* in the OE and SE lines.
**Table S1** Promoter motifs of genes mediating abiotic stress responses and lignin biosynthesis regulated by BplMYB46.
**Table S2** Primer sequences used in the construction of pROK2‐*BplMYB46*‐GFP.
**Table S3** Primer sequences used to amplify the whole or truncated CDS of *BplMYB46* in the transactivation assay.
**Table S4** Primer sequences of genes analyzed by real‐time RT‐PCR.
**Table S5** Primer sequences used in the Y1H assay.
**Table S6** Primer sequences used in the construction of the reporter constructs analyzed in tobacco plants.
**Table S7** Primer sequences used in the construction of the reporter constructs to verify the results of the ChIP assay.
**Table S8** Primer sequences used in the construction of *BplMYB46* overexpression and silencing lines.
**Table S9** Primer sequences used in the analysis of *BpMYB61* expression in *BplMYB46* overexpression and silencing lines.
**Table S10** Primer sequences used in the analysis of BplMYB46 target genes using real‐time RT‐PCR.
**Table S11** Primer sequences used in ChIP‐PCR analysis.
