# Supplementary material for: Expression of the MYB transcription factor gene BplMYB46 affects abiotic stress tolerance and secondary cell wall deposition in Betula platyphylla
Source: Plant Biotechnol J. 2016 Aug 1;15(1):107–21. doi: 10.1111/pbi.12595 (PMC5253473; doi:10.1111/pbi.12595)
Supplement: Supplementary file 1 — Figure S1 Sequence alignments of BplMYB46 with other plant MYBs. Figure S2 Phylogenetic comparison of BplMYB46 with other plant MYBs. Figure S3 Relative expression of BplMYB46 in the OE and SE lines. Table S1 Promoter motifs of genes mediating abiotic stress responses and lignin biosynthesis regulated by BplMYB46. Table S2 Primer sequences used in the construction of pROK2‐BplMYB46‐GFP. Table S3 Primer sequences used to amplify the whole or truncated CDS of BplMYB46 in the transactivation assay. Table S4 Primer sequences of genes analyzed by real‐time RT‐PCR. Table S5 Primer sequences used in the Y1H assay. Table S6 Primer sequences used in the construction of the reporter constructs analyzed in tobacco plants. Table S7 Primer sequences used in the construction of the reporter constructs to verify the results of the ChIP assay. Table S8 Primer sequences used in the construction of BplMYB46 overexpression and silencing lines. Table S9 Primer sequences used in the analysis of BpMYB61 expression in BplMYB46 overexpression and silencing lines. Table S10 Primer sequences used in the analysis of BplMYB46 target genes using real‐time RT‐PCR. Table S11 Primer sequences used in ChIP‐PCR analysis. [file PBI-15-107-s001.docx]

**Table S1 Promoter motifs of genes mediating abiotic stress responses and lignin biosynthesis regulated by *BplMYB46***

| Promoter  0— -1100bp | GenBank number of genes | Motifs |
| --- | --- | --- |
| SOD4 | KP711291 | MYBCORE, AC-box |
| SOD6 | KP711293 | MYBCORE |
| POD3 | KP711298 | MYBCORE |
| POD4 | KP711299 | MYBCORE, AC-box |
| POD9 | KP711304 | MYBCORE, AC-box |
| POD10 | KP711305 | MYBCORE, AC-box |
| P5CS1 | KP711294 | MYBCORE, AC-box |
| P5CS2 | KP711295 | MYBCORE |
| PAL1 | KP711309 | MYBCORE |
| PAL2 | KP711310 | MYBCORE, AC-box |
| CCoAOMT1 | KP711311 | MYBCORE |
| CCoAOMT2 | KP711312 | MYBCORE, AC-box |
| 4CL2 | KP711314 | MYBCORE, AC-box |
| LAC1 | KP711316 | MYBCORE, AC-box |
| CESA 1 | KP711317 | MYBCORE |
| CESA 3 | KP711319 | MYBCORE, AC-box |
| MYB1 | KP711285 | MYBCORE |
| MYB3 | KP711287 | MYBCORE, AC-box |

**Table S2 Primer sequences used in the construction of pROK2-BplMYB46-GFP**

| Primer | Sequence (5’-3’) |
| --- | --- |
| MYB-F | CTCTAGAGGATCCCCATGAGGAAGCCGGAGGCCT |
| *BplMYB46-*GFP-R | CTGAACTTGGAAATCAAGT |
| GFP*-*R | TCGAGCTCGGTACCCTCACTTGTACAGCTCATCC |
| pROK2-F | CTCCACTGACGTAAGGGAT |
| pROK2-R | CGCAAGACCGGCAACAGGAT |

**Table S3 Primer sequences used to amplify the whole or truncated CDS of *BplMYB46* in the transactivation assay.**

| Primer | Sequence (5’-3’) |
| --- | --- |
| BD-MYB.F | CATGGAGGCCGAATTCATGAGGAAGCCGGAGGCCT |
| BD-MYB.R | GCAGGTCGACGGATCCCTGAACTTGGAAATCAAG |
| BD1-F | CATGGAGGCCGAATTCATGAGGAAGCCGGAGGCCT |
| BD1-R | GCAGGTCGACGGATCCCATATACATCATGGGCATC |
| BD2-F | CATGGAGGCCGAATTCGATTCATCATCATCATCAT |
| BD2-R | GCAGGTCGACGGATCCCTGAACTTGGAAATCAAG |
| BD3-F | CATGGAGGCCGAATTCATGAGGAAGCCGGAGGCCT |
| BD3-R | GCAGGTCGACGGATCCCATGTTAAGGGCGCCATGC |
| BD4-F | CATGGAGGCCGAATTCTCAACCGGATATGATCTT |
| BD4-R | GCAGGTCGACGGATCCCTGAACTTGGAAATCAAG |
| BD5-F | CATGGAGGCCGAATTCATGAGGAAGCCGGAGGCCT |
| BD5-R | GCAGGTCGACGGATCCAAACCCAAAGATCCCAT |
| BD6-F | CATGGAGGCCGAATTCGATGGGGACCTGTTTGTT |
| BD6-R | GCAGGTCGACGGATCCCTGAACTTGGAAATCAAG |
| BD7-F | CATGGAGGCCGAATTCGATGGGGACCTGTTTGTT |
| BD7-R | GCAGGTCGACGGATCCATTTCCAATCCGAGCTAAGT |
| BD8-F | CATGGAGGCCGAATTCTATTGGGAAGTAGAAGAGTT |
| BD8-R | GCAGGTCGACGGATCCCTGAACTTGGAAATCAAG |

**Table S4 Primer sequences of genes analyzed by real-time RT-PCR.**

| Primer | Sequence (5’-3’) |
| --- | --- |
| *MYB-s* | TCAGGTGGAGGTGAGAAA |
| *MYB-r* | CAAGAAGGGAGTGCAAAT |
| Ubiquitin F | GGAGGACAAGGTGGAGGG |
| Ubiquitin R | GATTGAGGGGAGGGATGC |
| α-tubulin F | TGGCTCGAATGCACTGTTGG |
| α-tubulin R | TCAACCGCCTTGTCTCTCAGG |

**Table S5 Primer sequences used in the Y1H assay.**

| Primer | Sequence (5’-3’) |
| --- | --- |
| MYB46-AD-Rec2-F | TGGCCATTATGGCCCGGGATGAGGAAGCCGGAGGCCTC |
| MYB46-AD-Rec2-R | GACATGTTTTTTCCCGGGCTGAACTTGGAAATCAAGT |
| pHIS2-F | GCCTTCGTTTATCTTGCCTGCTC |
| pHIS2-R | CGATCGGTGCGGGCCTCTTC |
| MYBCORE-F | AATTCCAGTTACAGTTACAGTTAGAGCT |
| MYBCORE-R | CTAACTGTAACTGTAACTGG |
| AC-box-F | AATTCACCAACTACCAACTACCAACTGAGCT |
| AC-box-R | CAGTTGGTAGTTGGTAGTTGGTG |

**Table S6 Primer sequences used in the construction of the reporter constructs analyzed in tobacco plants.**

| Primer | Sequence (5’-3’) |
| --- | --- |
| p1301-MYBCORE-F | AGCTTCAGTTACAGTTACAGTTAACCCTTCCTCTATATAAGGAAGTTCATTTCATTTGGAGAGAACACGGC |
| p1301-MYBCORE-R | CATGGCCGTGTTCTCTCCAAATGAAATGAACTTCCTTATATAGAGGAAGGGTTAACTGTAACTGTAACTGA |
| p1301-AC-box-F | AGCTTACCAACTACCAACTACCAACTACCCTTCCTCTATATAAGGAAGTTCATTTCATTTGGAGAGAACACGGC |
| p1301- AC-box-R | CATGGCCGTGTTCTCTCCAAATGAAATGAACTTCCTTATATAGAGGAAGGGTAGTTGGTAGTTGGTAGTTGGTA |
| p1301-mMYBCORE-F | AGCTTACACCCACACCCACACCCACCCTTCCTCTATATAAGGAAGTTCATTTCATTTGGAGAGAACACGGC |
| p1301-mMYBCORE-R | CATGGCCGTGTTCTCTCCAAATGAAATGAACTTCCTTATATAGAGGAAGGGTGGGTGTGGGTGTGGGTGTA |
| p1301-mAC-box-F | AGCTTCAACCACCAACCACCAACCACACCCTTCCTCTATATAAGGAAGTTCATTTCATTTGGAGAGAACACGGC |
| p1301-mAC-box-R | CATGGCCGTGTTCTCTCCAAATGAAATGAACTTCCTTATATAGAGGAAGGGTGTGGTTGGTGGTTGGTGGTTGA |

**Table S7 Primer sequences used in the construction of the reporter constructs to verify the results of the ChIP assay.**

| Primer | Sequence (5’-3’) | Length of truncated promoter (bp) |
| --- | --- | --- |
| SOD4-1301-F | GCAGGCATGCAAGCTTCGTGAGCTATTGAGCTTTTAC | 240 |
| SOD4-1301-R | GAGGAAGGGTAAGCTTAACGTTACTTTATCGTTATTT |  |
| POD3-1301-F | GCAGGCATGCAAGCTTTAAATGTTATCCTATTAAAT | 249 |
| POD3-1301-R | GAGGAAGGGTAAGCTTGACTTGATGGGTATAAGATAT |  |
| P5CS1-1301-F | GCAGGCATGCAAGCTTTAAAAAAATATATATATATAT | 243 |
| P5CS1-1301-R | GAGGAAGGGTAAGCTTGGGGCCCACCTCTCAATGAAAG |  |
| PAL2-1301-F | GCAGGCATGCAAGCTTTGTAATGCGTACCATTACTTC | 246 |
| PAL2-1301-R | GAGGAAGGGTAAGCTTCGATATTGTGGCAGAGGTTT |  |
| Cco1-1301-F | GCAGGCATGCAAGCTTACTGAGTTTTCTTTTGATGAAT | 234 |
| Cco1-1301-R | GAGGAAGGGTAAGCTTATTCATCAAAAGAAAACTCAGT |  |
| 4CL2-1301-F | GCAGGCATGCAAGCTTCCCTCAAAATAACAAGAAAAAT | 246 |
| 4CL2-1301-R | GAGGAAGGGTAAGCTTGGTAGTTGGTGGAGGGACTT |  |
| LAC1-1301-F | GCAGGCATGCAAGCTTCACTATGATACTGTTAAACT | 246 |
| LAC1-1301-R | GAGGAAGGGTAAGCTTCGTGACATATACAACAGGATAC |  |
| CESA3-1301-F | GCAGGCATGCAAGCTTGTTCAGCTCTTGGGATCTGT | 234 |
| CESA3-1301-R | GAGGAAGGGTAAGCTTCCACAGTCATCAATTGCTCC |  |
| MYB3-1301-F | GCAGGCATGCAAGCTTGAAGCAAGAAGGCAGATGTC | 237 |
| MYB3-1301-R | GAGGAAGGGTAAGCTTTCCAAAAACCTTATCTAAAGC |  |

**Table S8 Primer sequences used in the construction of *BplMYB46* overexpression and silencing lines.**

| Primer | Sequence (5’-3’) |
| --- | --- |
| MYB-F | CTCTAGAGGATCCCCATGAGGAAGCCGGAGGCCT |
| MYB-R | TCGAGCTCGGTACCCTTACTGAACTTGGAAATCAAG |
| promoter-Cis-F | CATGCCATGGCATGGATAATGTGAAGTGGAAGC |
| promoter-Cis-R | CATGATTTAAATCGACCTGTGTGTTTGTGTGT |
| promoter-Anti-F | CGGGATCCCGACCTGTGTGTTTGTGTGT |
| promoter-Anti-R | GCTCTAGAGCGATAATGTGAAGTGGAAGC |
| p5941-Cis-F | CGCTCGAGTATAAGAGCT |
| p5941-Cis-R | ACCTTCCCACAATTCGTCGG |
| p5941-Anti-F | GCATGCTATGCATTCAAT |
| p5941-Anti-R | CGTGCACAACAGAATTGAAAGC |

**Table S9 Primer sequences used in the analysis of *BpMYB61* expression in *BplMYB46* overexpression and silencing lines*.***

| Primer | Sequence (5’-3’) |
| --- | --- |
| MYB61-F | TAAGGAGACCCAAACCC |
| MYB61-R | TGTGCCCACATAGCAGT |

**Table S10 Primer sequences used in the analysis of BplMYB46 target genes using real-time RT-PCR.**

| Primer | Sequence (5’-3’) | GenBank ID |
| --- | --- | --- |
| SOD1-F | CCGTGGTTGTTCTTGGCAAC | KP711288 |
| SOD1-R | CCAGCAGGATTGAAATGTGGC |  |
| SOD2-F | TGCTCCTAAAGCGGTGGTTC | KP711289 |
| SOD2-R | GGGTTGAAATGAGGGCCAGT |  |
| SOD3-F | GAAGCCGCACTAGTAGCCAT | KP711290 |
| SOD3-R | ACGACAGTGAGTGGTTTGGC |  |
| SOD4-F | CGGAGGTCATATCAACCACTC | KP711291 |
| SOD4-R | CAGACCAAGCCACACCCAT |  |
| SOD5-F | TCTGGAAGAATCTCGCACCC | KP711292 |
| SOD5-R | ATCAAGAGCAAGCCAAACCC |  |
| SOD6-F | CTTTGCTCTTTCCTCACTCT | KP711293 |
| SOD6-R | ACAACCCTCGCATTTCAT |  |
| POD1-F | ACTAAATCTACCACCACCC | KP711296 |
| POD1-R | TGTATCGCCCTTTCCAG |  |
| POD2-F | AGCATAAAAGAGCATCCCTC | KP711297 |
| POD2-R | CACATCCAGACCCAGAAATT |  |
| POD3-F | TTCATTGCGAGGGTTTG | KP711298 |
| POD3-R | TGCTTGAGAAGGCAGATAG |  |
| POD4-F | TACTGGACGCAAAGATGG | KP711299 |
| POD4-R | AGGAAGGAAAGCGGAAT |  |
| POD5-F | TGAGGTGAATGGGAAGAATG | KP711300 |
| POD5-R | AGGTGATGTGGTTGGTGC |  |
| POD6-F | CAAGTCGGCAGTTGAGAAT | KP711301 |
| POD6-R | TGGAAAGGCCCACATTAC |  |
| POD7-F | ACATCTTAGCCGTTGTTGC | KP711302 |
| POD7-R | GGCAGTGAGACCTTTGTTT |  |
| POD8-F | TGTCAACAATTCTGGGCTAC | KP711303 |
| POD8-R | CCACCCTACAGTTCTTCCT |  |
| POD9-F | AGGAACGAGAACGCTACTGC | KP711304 |
| POD9-R | AACACGATCCGATCTGATGC |  |
| POD10-F | GATGGCAGGGTATCTTTG | KP711305 |
| POD10-R | GCTTGGTTTATGGTGGG |  |
| P5CS1-F | CAAACGCCACCTCACAGAC | KP711294 |
| P5CS1-R | TCGTGTAACAACGGCAGTCC |  |
| P5CS2-F | TGGGAACGGTCTACTGCTT | KP711295 |
| P5CS2-R | TTACTGCCTCTTGGGATTAC |  |
| P5CDH1-F | GACTTCAGCCTCCAACACCT | KP711306 |
| P5CDH1-R | TCAACCAGCCTAACCAACC |  |
| P5CDH2-F | TTCTATACCCTTTGCTACCG | KP711307 |
| P5CDH2-R | GAATGCCTGTTTCATCTACC |  |
| ProDH-F | GAGTACACTTCCATCCAACC | KP711308 |
| ProDH-R | ATGCTCCCCTCACCAAT |  |
| Pal1-F | GAGGCAAAATGTGGTAGC | KP711309 |
| Pal1-R | CATCATCTTTCCCGTCC |  |
| Pal2-F | TTTTGTCGGAGATCCTGTC | KP711310 |
| Pal2-R | AAGCCATTGCGGTGAAG |  |
| Cco1-F | GACATTGGTCGGAAGCAG | KP711311 |
| Cco1-R | TGAGGAAGAGTAAGGGGTT |  |
| Cco2-F | TTTCACTCTATCCCTTCTGC | KP711312 |
| Cco2-R | CTCCCATTCTTTCTGGTCTC |  |
| Cco3-F | GCCTGAACCCATGAAGGAGC | KT223488 |
| Cco3-R | TGTGGCGAGGAGGGAGTAGC |  |
| Cco4-F | ACCTCAGCCGATGAAGGAC | KT223489 |
| Cco4-R | TTTGAATGACAGGCAAACCC |  |
| 4CL1-F | GCGGGACTTTCCAATCT | KP711313 |
| 4CL1-R | AAGTTCTCGCCTGTTTCC |  |
| 4CL2-F | CCCATCCACTCCTATTGCT | KP711314 |
| 4CL2-R | CCGATTTCGCTTGCTTTG |  |
| 4CL3-F | GACACTGGCGTCAGGAAAGG | KP711315 |
| 4CL3-R | GCGAGTTGTGAAAGCGAGG |  |
| LAC1-F | AACCCTTGCTCTACTTGCG | KP711316 |
| LAC1-R | TTTTCGGCGGCTATCAT |  |
| LAC2-F | AACAGTTCACGGTGGCATAG | KT223490 |
| LAC2-R | GAGCATCGGAGACATTAGGC |  |
| CCR-F | ATCAGCAACTGGAAGGCACT | JQ783349 |
| CCR-R | CACCGGAATGAATTGTAAGC |  |
| CESA1-F | GAAGACATTGACGAGGGTG | KP711317 |
| CESA1-R | AAGAGTCGCAGGGTTGGTT |  |
| CESA2-F | ATCCACCCAGCGTCTTT | KP711318 |
| CESA2-R | TGCCTTCATCCATCGTG |  |
| CESA3-F | CTGCGGTGATAATGTTGG | KP711319 |
| CESA3-R | GACTTCCTTTGTGCCTCTT |  |
| FRA-F | GAACTTGAAATCGGACCTG | KU168419 |
| FRA-R | ATGGATGACACCAGCACTCT |  |
| IRX-F | ACTCTACGGTTGGTGGC | KU168420 |
| IRX-R | TATGGTGCTCGATGTGC |  |
| MYB1-F | GCGTCTTGGCAATAGGT | KP711285 |
| MYB1-R | TGATGCTGTCGTTTTCG |  |
| MYB2-F | TTGCCGCCATTGATAGAG | KP711286 |
| MYB2-R | CCCACATTTGACGAAGC |  |
| MYB3-F | GATTTGCCTACCCCTTC | KP711287 |
| MYB3-R | AACACCAGCCTTGTTCTGC |  |

**Table S11 Primer sequences used in ChIP-PCR analysis**

| Primer | Sequence (5’-3’) | Length of amplification (bp) |
| --- | --- | --- |
| SOD4-F | TAACTGGCAATTGCACTTT | 136 |
| SOD4-R | CATTGATGGAAAGGCAATT |  |
| SOD6-F | GTCACTCGCCATTGTTTCT | 174 |
| SOD6-R | GCAAAATCCTTACTAACCGT |  |
| POD3-F | ATATGTCATATTAACAAGAA | 196 |
| POD3-R | TTGATGGGTATAAGATATGTT |  |
| POD4-F | GAGACATGTGCATTAATAGC | 179 |
| POD4-R | AGAGCAGTGGCTGGGCTCTT |  |
| POD9-F | GACTTTCAACGAGCATCCGC | 160 |
| POD9-R | TAGTTGACCTTACTTACCAT |  |
| POD10-F | ACCAAATCAAACAGATTTAATT | 196 |
| POD10-R | ATGTAGCTAGGGTAATAATG |  |
| P5CS1-F | CTCAATGAGTATGAGAGGG | 192 |
| P5CS1-R | TCAATGAAAGGTGGGCCCC |  |
| P5CS2-F | TAATTGAGATTTTATATTTC | 173 |
| P5CS2-R | TGGCAACTGAAGGAGTTCGC |  |
| PAL1-F | TCTAGCAGAGGAGAAAGAGG | 199 |
| PAL1-R | CAACGAAGGTGCTATCAAGC |  |
| PAL2-F | CGGAGTAAAGAGAATCATTG | 152 |
| PAL2-R | CGATATTGTGGCAGAGGT |  |
| Cco1-F | GCATTTTTTTATCCACCTGT | 167 |
| Cco1-R | TCATAGTCTCTTTATTTATTT |  |
| Cco2-F | ATGGCCCACACAGGCCCCT | 175 |
| Cco2-R | AGATTTATGGATTCATCCC |  |
| 4CL2-F | GGTAGGTGAAGAAGACACTG | 199 |
| 4CL2-R | GTTGGTGGAGGGACTTAACG |  |
| LAC1-F | GGTTTAATTAATATTTTAAC | 185 |
| LAC1-R | CATATACAACAGGATACCAC |  |
| CESA1-F | GGCAGGGGCTAATTCAGTCCG | 199 |
| CESA1-R | ATCTAGAGAATTCAGCTCAT |  |
| CESA3-F | GCCTTAGAATTAAGATTTGG | 150 |
| CESA3-R | CTCCTGTAAATGGAAATTTC |  |
| MYB1-F | CTTTTGGACTTAAAAAATT | 166 |
| MYB1-R | TTGCTTATATAAAAAAAAAAT |  |
| MYB3-F | GTGATCAGCTTTCTAAGC | 197 |
| MYB3-R | CCAAAAACCTTATCTAAAGC |  |
| α-tubulin-F | GCATTCTGATGCCATTTC | 191 |
| α-tubulin-R | CAAGAAGGCCGCCACCAG |  |

**Figure. S1**

**
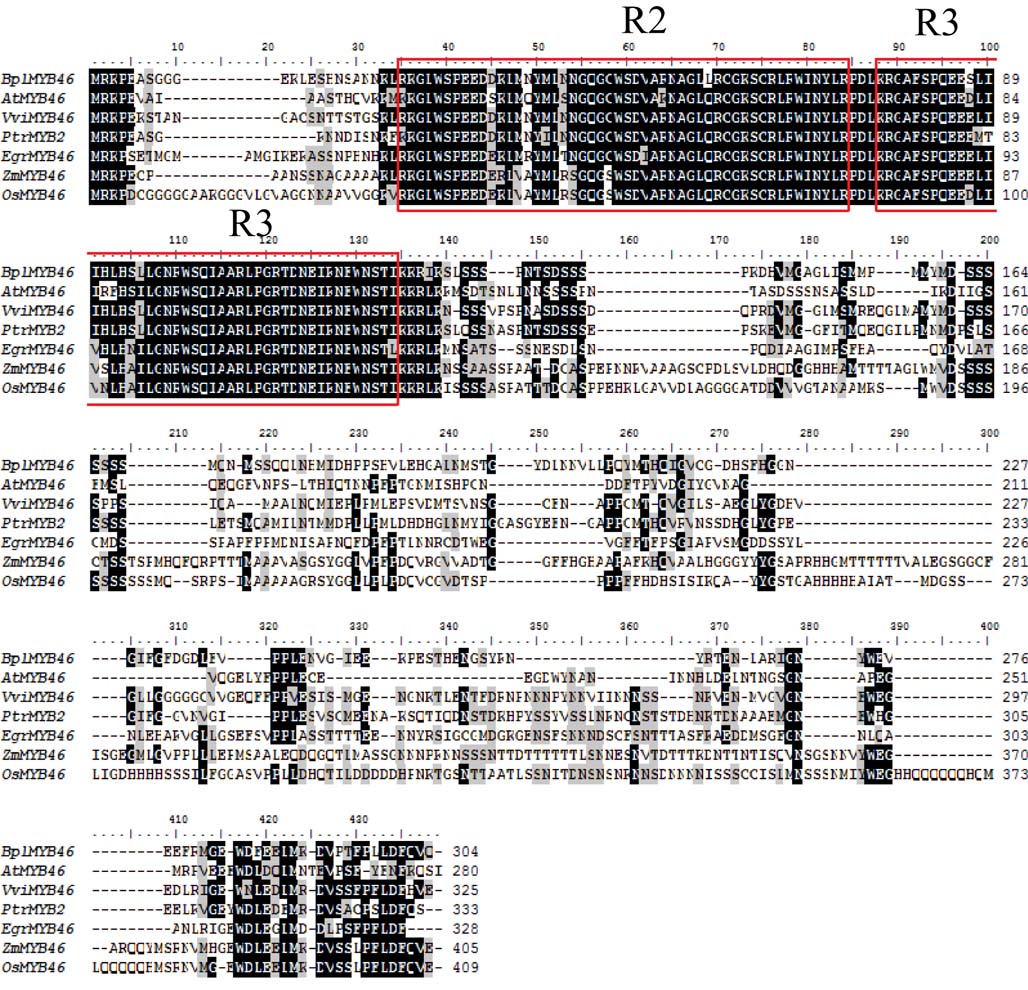
**

**Sequence alignments of BplMYB46 with other plant MYBs.** Alignments of BplMYB46 with other MYBs from various plants; the red boxes marked by R denote conserved amino acid residues. GenBank accession numbers: BplMYB46: KP711284, AtMYB46 (*Arabidopsis thaliana*): AED91824, VviMYB46 (*Vitis vinifera*): XM_002275431, PtrMYB2 (*Populus trichocarpa*): AGT02397, EgrMYB46 (*Eucalyptus grandis*): XP_010067873, ZmMYB46 (*Zea mays*): NP_001241859, OsMYB46 (*Oryza sativa*): AEO53060.

**Figure. S2**

**
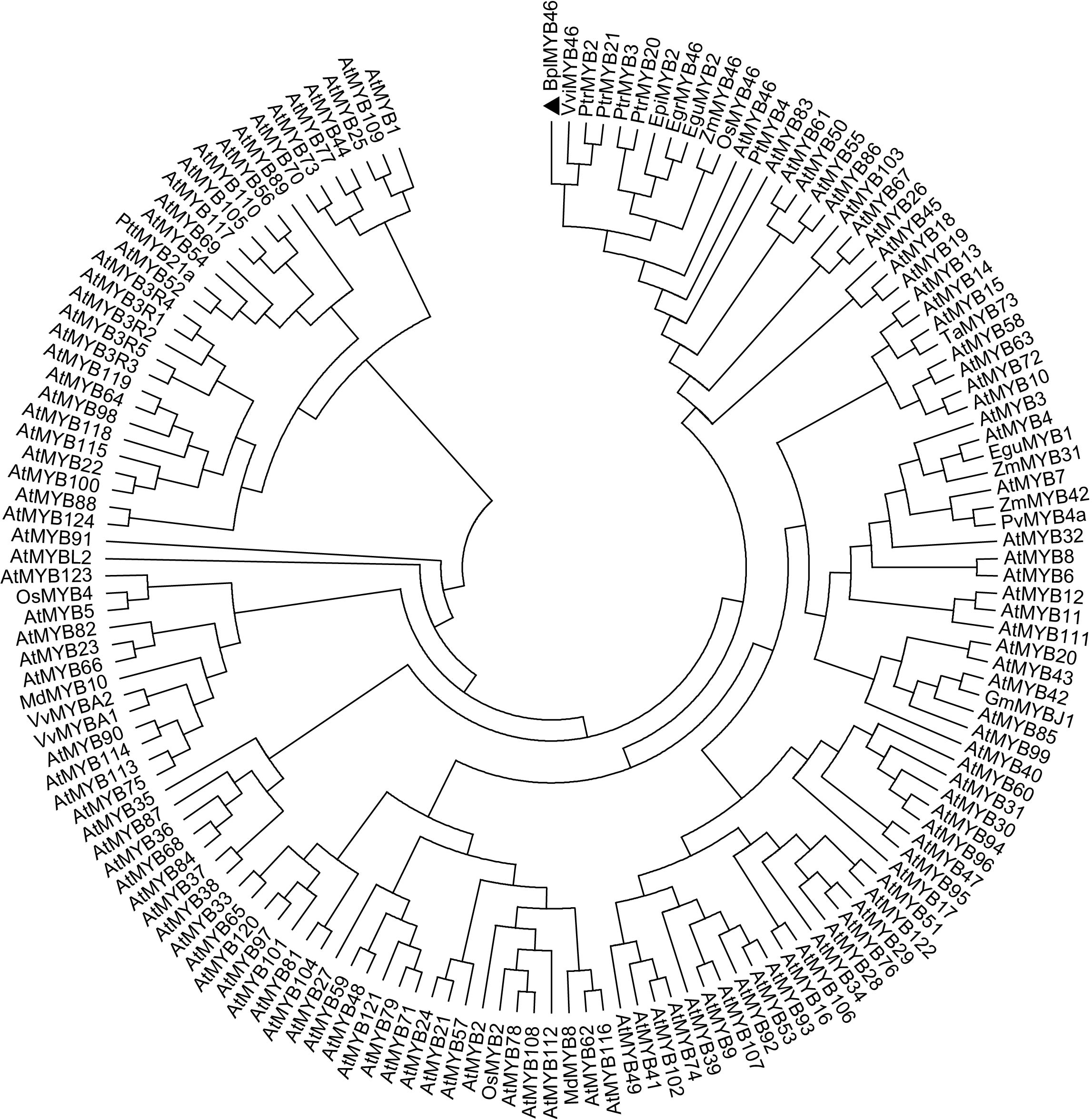
**

**Phylogenetic comparison of BplMYB46 protein with other plant MYBs.** Phylogenetic tree of BplMYB46 compared with MYBs from various plant species. The amino acid sequences were retrieved from GenBank: VviMYB46 (*Vitis vinifera*): XM_002275431. PtrMYB2, MYB3, MYB20, MYB21 (*Populus trichocarpa*): AGT02397, AGT02395, AGT02396, AGT02398. EpiMYB2 (*Eucalyptus pilularis*): BAM05600. EgrMYB46 (*Eucalyptus grandis*): XP_010067873. EguMYB1, MYB2 (*Eucalyptus gunnii*): CAE09058, CAE09057. Zm MYB31, MYB42, MYB46 (*Zea mays*): NP_001105949, NP_001106009, NP_001241859. OsMYB2, 4, MYB46 (*Oryza sativa*): BAA23338, AAP54284, AEO53060. PtMYB4 (*Pinus taeda*): AAQ62540. TaMYB73 (*Triticum aestivum*): AEW23186. PvMYB4a (*Panicum virgatum*): AEM17348. GmMYBJ1 (*Glycine max*): AGO06072. MdMYB8, 10 (*Malus domestica*): ABB84756, BAJ24837. PttMYB21a (*Populus tremula* x *Populus tremuloides*): CAD98762. VvMYBA1, 2 (*Vitis vinifera* MYB-related transcription factor): BAD18977, BAD18978. All *Arabidopsis thaliana* MYB transcription factor sequences were obtained from the *Arabidopsis* database (<http://www.arabidopsis.org/>).

**Figure. S3**

**
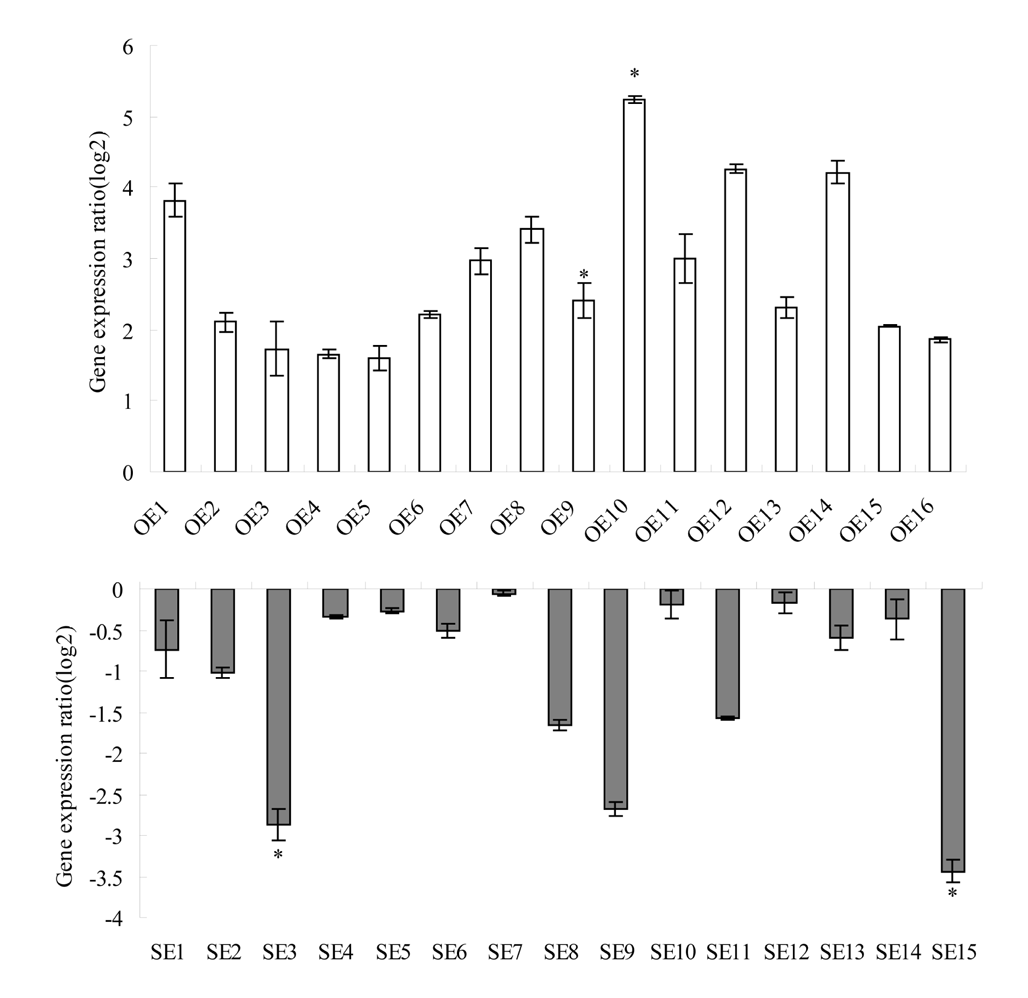
**

**Relative expression of *BplMYB46* in the OE and SE lines.** Relative expression of *BplMYB46* in transgenic lines overexpressing *BplMYB46* (OE) and RNAi-silenced *BplMYB46* (SE) lines. Sixteen OE lines and fifteen SE lines were examined. The lines marked with [asterisk](javascript:void(0);)s were used for further study.
